# Age-structured mechanical models for tumor growth

**DOI:** 10.1007/s00285-026-02389-z

**Published:** 2026-04-06

**Authors:** Doron Levy, Hyunah Lim, Antoine Mellet, Maeve Wildes

**Affiliations:** Department of Mathematics, University of Maryland College Park, College Park, MD 20742 USA

**Keywords:** Tumor growth, Age-structured model, Nonlinear Darcy’s law, Cross-diffusion, Existence of solutions, 35Q92, 92-10, 35G61

## Abstract

In this paper, we introduce and analyze a mechanical model for tumor growth that takes into account the life cycle of a tumor cell. The underlying process for tumor growth is the same as in classical mechanical models: the spatial expansion of the tumor is driven by the proliferation of the cells (mitosis) which is only limited by the pressure inside the tissue. The natural incompressibility of the cells, which leads to a movement of the cells away from regions of high pressure, is taken into account via a nonlinear Darcy’s law. Compared to similar models studied recently, we include an additional variable, which represents the age of the cells. The various phases of the life of a cell (growth, mitosis and death) are then dependent on this age variable. We prove the existence of weak solutions and investigate their behavior numerically, focusing on the age distribution of the cells inside the tumor, the convergence to traveling wave solutions and the existence of a threshold for the death rate for expansion/regression of the tumor.

## Introduction

### An age-structured mechanical model

Numerous mathematical models for tumor growth have been developed and studied. Typically such models take into account several key mechanisms of tumor invasion, such as competition for space (Brú et al. 2003; Byrne and Drasdo 2009; Byrne and Preziosi 2003; Preziosi and Tosin 2009) (with other cancer cells as well as with healthy cells), the availability of nutrients (Greenspan 1972; Chaplain 1996; Perthame et al. 2014a), phenotypic traits that might affect a cell’s behavior (Lorenzi et al. 2021; Fiona R Macfarlane et al. 2022), etc. In this paper, we focus on the simplest type of mechanical models in which the growth of the tumor is driven by the proliferation of the cells, which is only limited by the pressure inside the tissue, and by Darcy’s law, which describes the movement of the cells away from regions of high pressure (Greenspan 1972; Sherratt et al. 2001; Tiina Roose et al. 2007; Cristini et al. 2009; Lowengrub et al. 2010). A classical model, introduced in Byrne and Drasdo (2009) (see also Brú et al. (2003); Byrne and Chaplain (1996); Byrne and Preziosi (2003)) and studied for example in Perthame et al. (2014a); Mellet et al. (2017); Degond et al. (2020); Chertock et al. (2019); Motsch and Peurichard (2018) models the evolution of the cell population distribution function *u*(*x*, *t*) by the nonlinear diffusion equation

1.1 $$\begin{aligned} \partial _t u - {\textrm{div}\,}(u \nabla p) = u F(p), \qquad p =\frac{m}{m-1}\left( \frac{u}{u_M}\right) ^{m-1}, \end{aligned}$$

where the growth rate *F*(*p*) is a decreasing function of the pressure which vanishes for some $p=p_M$ (this maximal pressure $p_M$ is called the homeostatic pressure). Equation (1.1) is a classical nonlinear degenerate diffusion equation known as the porous media equation (Friedman 1983; Vazquez 2006). In the limit $m\rightarrow \infty$, the pressure vanishes whenever $u<u_M$ and goes to infinity when $u>u_M$. This singular limit has the effect of enforcing a maximum density $u\le u_M$ (see Perthame et al. (2014a)). Even for finite $m\gg 1$, we can think of $u_M$ as a threshold above which the effect of the pressure dominates the dynamic. In what follows, we take this threshold to be 1 (to simplify the notations) and we fix $m>1$.

The main object of this paper is a mechanical model for tumor growth which is based on the same simple considerations as (1.1), but which takes into account the life cycle of the cancer cells. The cell’s cycle (growth, DNA synthesis, mitosis) is a critical process essential for normal cell function and transitions between phases are controlled by complex mechanisms. The break down of these mechanisms leads to uncontrolled proliferation and genetic instability which are key features of cancer (Hanahan et al. 2000; Negrini et al. 2010). The cell’s cycle also plays a key role in the development of therapies; for instance, an accurate description of the distribution of cells in their mitosis phase is critical to the improvement of therapies based on anti-mitotic drugs (Lorz et al. 2018). For these reasons, it is essential to develop mathematical models that account for the different phases either by compartmentalizing the population according to the cells cycle phase (Giulia et al. 2021; Falcó et al. 2025) or by structuring the model with an age variable (Gyllenberg and Webb 1987; Dyson et al. 2002; Gabriel et al. 2012; Liu et al. 2018; Luo et al. 2023). In this paper, we adopt this second approach and focus on the role of two main phases of the cell’s life cycle: In the first phase, the so-called *interphase*, the cell grows and copies its genetic material. During the second phase, the *mitotic phase*, it splits into two daughter cells which can start the cycle from the beginning. We associate an age to each individual cell, denoted by $\theta \ge 0$, which we define for now as the time since its last mitosis. We then introduce the cell distribution function $n(x,\theta ,t)$ which can be interpreted as the probability of finding a cell of age $\theta \ge 0$ at a position $x\in \mathbb {R}^d$. The evolution of this distribution function under pressure forces and cell duplication is described by the following boundary value problem:

1.2 $$\begin{aligned} {\left\{ \begin{array}{ll} \partial _t n + \partial _{\theta } n - {\textrm{div}\,}_{x} (n \nabla _{x} p) = - \nu (\theta ,p) \, n - \mu (\theta ) n \qquad & x\in \mathbb {R}^{d},\; \theta>0, \; t>0\\ \displaystyle n(x,0,t) = 2 \int _0^{\infty } \nu (\theta ,p)\, n(x,\theta ,t) \, d\theta & x\in \mathbb {R}^{d},\; \; t>0\\ n(x,\theta ,0)= n_{in}(x,\theta ) & x\in \mathbb {R}^{d},\; \theta >0, \end{array}\right. } \end{aligned}$$

where the initial data $n_{in}(x,\theta )$ describes the state of the tumor at $t=0$. In this equation, the term $\partial _\theta n$ accounts for the aging of a cell and the coefficient $\nu$ can be interpreted as the probability that a given cell enters its mitotic phase. When a cell enters its mitotic phase, that cell is lost (the term $- \nu n$ in the right hand side of the first equation), but two new cells, of age $\theta = 0$, are created (which gives rise to the boundary condition in (1.2)). In general, $\nu$ depends on both the age of the cell $\theta$ and the local pressure $p=p(x,t)$. We note that in experiments, there appears to be a lot of variability in the typical time between two mitosis events (Gabriel et al. 2012) so $\nu (\theta ,p)$ (which can be interpreted as the proportion of cells of age $\theta$ that will duplicate during one unit of time) will be non-zero for a large range of values of $\theta$. Pressure-limited proliferation corresponds to the assumptions

1.3 $$\begin{aligned} \partial _p \nu (\theta ,p) \le 0, \qquad \nu (\theta ,p)=0 \quad \text{ for } p\ge p_M. \end{aligned}$$

The coefficient $\mu (\theta )$, usually called the death rate, can be a constant or a function of the age of the cell. A maximum cell age $\theta _m$ can be imposed by requiring that $\int _0^{\theta _{m}} \mu (\theta )\, d\theta =+\infty$. We should stress out the fact that the term $-\mu (\theta ) n$ in the equation describes the physical removal of (dead) cells from the tumor, which creates space for new cell growth. Apoptosis (the death of cells) can also be taken into account by assuming that $\nu (\theta ,p)=0$ for $\theta \ge \theta _1$. In that case, cells of age $\theta >\theta _1$ will no longer proliferate and can be considered as inactive, but are still physically present in the tumor (they take up space thus preventing new cells from being born).

As in (1.1), the redistribution of the cells away from crowed regions is modeled by Darcy’s law, but the pressure *p* now depends not on the local “number" of cells $u(x,t)=\int _0^\infty n(x,\theta ,t)\, d\theta$ but on the volume occupied by the cells. Indeed, we wish to take into account the first phase of the cells life cycle (growth) by assuming that a cell of age $\theta \ge 0$ occupies a volume $V(\theta )$, where $\theta \mapsto V(\theta )$ is a non-decreasing function. The volume density is then defined as

$$\begin{aligned} \rho (x,t) = \int _0^\infty V(\theta ) n(x,\theta ,t)\, d\theta . \end{aligned}$$

Finally, the pressure is an increasing function of the volume density $\rho$, and as in (1.1) we can take a simple power law:

$$\begin{aligned} p(x,t) = \frac{m}{m-1} \rho (x,t) ^{m-1}, \end{aligned}$$

with parameter $m>1$ and maximum packing density $\rho _M=1$.

The purpose of this paper is to prove the existence of weak solutions for (1.2) and to investigate some important properties of the model numerically. We note that the uniqueness for such cross-diffusion model is a challenging and largely open problem and will not be addressed in this paper. Our analysis can be extended to some related models that take into account other phenomena that we have ignored here. We mention some of those below.

**Pressure-limited growth.** In (1.2), we assumed (see (1.3)) that high pressure (or high volume density) prevents the cells from dividing, thus limiting the growth of the tumor. Alternatively, we can assume that high pressure will slow down the whole life cycle of the cell (potentially preventing a newborn cell from growing and reaching its “adult" size). To write such a model, we can change slightly the meaning of the variable $\theta$, to represent a parameter indicating how far along its life cycle a cell has been able to go (i.e. the cell physiological age). In this model, a cell no longer “ages" linearly in time, since its growth is limited by a parameter depending on the pressure *p*, leading to the system:

1.4 $$\begin{aligned} {\left\{ \begin{array}{ll} \partial _t n + r(p) \partial _{\theta } n - {\textrm{div}\,}_{x} (n \nabla p) = - r(p) \nu (\theta ,p) n - \mu (\theta ) n \qquad & x\in \mathbb {R}^{d},\; \theta>0, \; t>0\\ \displaystyle n(x,0,t) = 2 \int _0^{\infty } \nu (\theta ,p) n(x,\theta ,t) \, d\theta & x\in \mathbb {R}^{d},\; \; t>0\\ n(x,\theta ,0)= n_{in}(x,\theta ) & x\in \mathbb {R}^{d},\; \theta >0, \end{array}\right. } \end{aligned}$$

where *r*(*p*) is a decreasing function of *p* such that $r(0)>0$ and $r(p) =0$ for $p\ge p_M$.

**The role of quiescent cells.** An important feature of many tumor growth models is the fact that not all cells behave similarly and that one should make a distinction between the proliferating cells, with distribution function still denoted by $n(x,\theta ,t)$ and the quiescent cells, with distribution function $q(x,\theta ,t)$ (Gyllenberg and Webb 1987; Dyson et al. 2002; Liu et al. 2018). After mitosis, a fraction $\lambda$ of the new cells will be quiescent (and the rest proliferating). In addition, cells can switch back and forth from one state to the other with probabilities $\sigma _1$ and $\sigma _2$. This leads to the following system of equations:

1.5 $$\begin{aligned} {\left\{ \begin{array}{ll} \partial _{t} n + r_{1}(p)\partial _{\theta } n - {\textrm{div}\,}_{x} (n \nabla _{x} p) = - \mu (\theta ) n - r_{1}(p)\nu (\theta ,p) n + \sigma _{1} q - \sigma _{2} n\\ \partial _{t} q + r_{2}(p) \partial _{\theta } q - {\textrm{div}\,}_{x} (q \nabla _{x} p) = -\mu (\theta ) q -\sigma _1 q + \sigma _2 n \\ \displaystyle n(x,0,t) = 2 (1-\lambda ) \int _{0}^{\infty } \nu (\theta ,p) n(x,\theta ,t) \, d\theta \\ \displaystyle q(x,0,t) = 2 \lambda \int _{0}^{\infty } \nu (\theta ,p) n(x,\theta ,t) \, d\theta , \\ \end{array}\right. } \end{aligned}$$

with $r_1$ and $r_2$ the different “aging speeds" for the two types of cells and

$$ p(x,t) = \frac{m}{m-1} \rho (x,t) ^{m-1}, \qquad \rho (x,t) = \int _0^\infty V_n(\theta ) n(x,\theta ,t)+ V_q(\theta ) q(x,\theta ,t)\, d\theta . $$

Here, $V_n(\theta )$ and $V_q(\theta )$ denote the (possibly different) volumes of the proliferating and quiescent cells. Note that the quiescent cells do not duplicate and might have a slower evolution if $r_1\ne r_2$. Taking $r_2(p)=0$ amounts to assuming that they have a frozen life cycle: they do not grow or duplicate - unless and until they transition to the proliferating state. The rates of transition between the proliferative and quiescent compartments, $\sigma _1$ and $\sigma _2$ can depend on the age variable $\theta$ as well as on the pressure (see Dyson et al. (2002) and reference therein).

### Equation for the density

Equations (1.2) and (1.4) are related to the classical model (1.1) as follows. When $\nu =\nu (p)$, $\mu =\mu _0$ and $V=V_0$ are all independent of the age $\theta$, we can integrate the first equation in (1.4) with respect to $\theta$ to get the following equation for $\rho (x,t)= V_0 \int _0^\infty n(x,\theta ,t)\, d\theta$:

1.6 $$\begin{aligned} {\left\{ \begin{array}{ll} \partial _t \rho - {\textrm{div}\,}(\rho \nabla p) = \rho \,( \nu (p) r(p)-\mu _0), \qquad p =\frac{m}{m-1} \rho ^{m-1} & x\in \mathbb {R}^d, \; t>0\\ \rho (x,0)= V_0 \int _0^\infty n_{in}(x,\theta ) \, d\theta & x\in \mathbb {R}^d, \end{array}\right. } \end{aligned}$$

which is (1.1) with growth rate $F(p) = \nu (p) r(p)-\mu _0$.

In this simple case, it is thus possible to solve for $\rho (x,t)$ without determining $n(x,\theta ,t)$. In general (when $\nu$, $\mu$ and *V* do depend on $\theta$), that is not possible: After multiplying the first equation in (1.4) by $V(\theta )$ and integrating with respect to $\theta$ we find the following equation for the volume density $\rho (x,t)$:

1.7 $$\begin{aligned} \begin{aligned} \partial _t \rho - {\textrm{div}\,}(\rho \nabla p)&=r(p) \int _0^\infty V'(\theta ) n(x,\theta ,t)\, d\theta \\&\quad + r(p) \int _0^\infty \nu (\theta ,p)(2V(0)-V(\theta )) n(x,\theta ,t)\, d\theta \\&\quad - \int _0^\infty \mu (\theta ) V(\theta ) n(x,\theta ,t)\, d\theta . \end{aligned} \end{aligned}$$

The first term in the right hand side describes the expansion of the tumor due to the volume change of individual cells, while the second term accounts for the change of volume during mitosis. Such events are typically volume preserving (i.e. the total volume of the two daughter cells is equal to the volume of the dividing cell), which can be enforced by assuming that $\nu (\theta ,\rho )(2V(0)-V(\theta )) = 0$ for all $\theta >0$. A simple form for the coefficients $\nu$ and *V* describing this situation is as follows:

1.8 $$\begin{aligned} V(\theta ) ={\left\{ \begin{array}{ll} V_0+ \alpha \theta & \text{ if } \theta \in [0,V_0/\alpha ]\\ 2 V_0 & \text{ if } \theta \ge V_0/\alpha ,\end{array}\right. } \end{aligned}$$

1.9 $$\begin{aligned} \nu (\theta ,p) = 0 \quad \text{ for } \theta <V_0/\alpha , \end{aligned}$$

in which case (1.7) reduces to

$$ \partial _t \rho -{\textrm{div}\,}(\rho \nabla p) = \alpha r(\rho ) \int _0^{V_0/\alpha } n(x,\theta ,t)\,d\theta - \int _0^\infty \mu (\theta ) V(\theta ) n(x,\theta ,t)\, d\theta . $$

### Motivations and related work

The fact that tumor cells of different ages have different proliferation and mortality rates is well-documented, and age-structured models for tumor cell population without spatial dependence have been investigated by many authors (Gyllenberg and Webb 1987; Dyson et al. 2002; Gabriel et al. 2012; Liu et al. 2018; Luo et al. 2023). A simple such model is the growth-fragmentation equation (M’Kendrick 1925; von Foerster 1959):

1.10 $$\begin{aligned} {\left\{ \begin{array}{ll} \partial _t n + \partial _{\theta } n = - \beta (\theta ) \, n - \mu (\theta ) n \qquad & \theta>0, \; t>0\\ \displaystyle n(0,t) = 2 \int _0^\infty \beta (\theta )\, n(\theta ,t) \, d\theta & \; t>0, \end{array}\right. } \end{aligned}$$

which corresponds to our model (1.2) in the space homogeneous case and without the pressure variable *p*.

Equation (1.10) can be used to predict the growth of the tumor and the evolution of the distribution of proliferating/quiescent cells in tumors. In Dyson et al. (2002), for example, Dyson et al. proved that the population eventually follows asynchronous exponential growth and that the final age distribution of the population is independent of the initial age distribution. Age-structure models have also been used to investigate the effects of the immune system, which plays a crucial role in protecting the body against tumors before they are large enough to be detected. The immune system detects and removes cancerous cells via a complex mechanism, but simplified mathematical models have been proposed (Luo et al. 2023; Pillis et al. 2005). In Luo et al. (2023), the following simple age-structured tumor immune model is analyzed:

1.11 $$\begin{aligned} {\left\{ \begin{array}{ll} \partial _t n + \partial _{\theta } n = - \beta (\theta ) \, n - \mu (\theta ) n -\sigma (\theta ) E(t) n\qquad & \theta>0, \; t>0\\ \displaystyle n(0,t) = 2 \int _0^\infty \beta (\theta )\, n(\theta ,t) \, d\theta & \; t>0, \end{array}\right. } \end{aligned}$$

where *E*(*t*) denotes the number of effector cells (immune cells responsible for identifying and clearing tumor cells), whose evolution is modeled by

$$\begin{aligned} \frac{d E(t)}{dt} = s-\mu _E E(t) + \tau N(t) E(t), \qquad N(t) = \int _0^\infty n(\theta ,t)\, d\theta . \end{aligned}$$

The existence and stability of steady states is investigated and the existence of a threshold for the existence of tumor-free steady state is established. Natural generalizations of (1.11) incorporate the role quiescent cells (Liu et al. 2018, 2019) and the role of nutrients (Liu et al. 2024). We also refer to Atsou et al. (2021) for a tumor immune model that takes into account the size of tumor cell in the cell division mechanism ($V(\theta )$ in our model).

In these models, the cell’s cycle is described by a continuous (age) variable $\theta >0$. A different class of mathematical models structures the population in compartments according to the main phases of the cell’s cycle (growth, DNA synthesis, mitosis, etc.) and describes the transition from one phase to another. We refer for example to Giulia et al. (2021); Falcó et al. (2025) and the review (Ma and Gurkan-Cavusoglu 2024) for further details on this different approach.

Most of the papers mentioned above do not, however, take into account the effect of the pressure on the proliferation rate nor the effect of the volume change during the first phase of the cell’s life cycle. Pressure plays a determinant role in the long time evolution of the tumor. Because increasing pressure will inhibit the proliferation of cells in the core of the tumor, only cells near the outer rim of the tumor contribute significantly to the growth of the tumor, leading to linear, instead of exponential, growth.

In Gurtin and MacCamy (1974, 1979), Gurtin and MacCamy study a nonlinear version of the growth fragmentation equation (1.10) which takes into account the number of cells to account for overcrowding effect. In the context of tumor growth, Liu et al. (2018) consider a nonlinear age-structured model in which the proliferation rate is a decreasing function of the number of tumor cells (which is a particular case of our model (1.5)). In these work, however, the migration of cells toward less crowded regions is not taken into account.

As mentioned in the introduction, other studies have focused on this migration and on the role of mechanical stress in the growth of the tumor (but without the age-structure) (Byrne and Drasdo 2009). The resulting models have been studied by the mathematical community for a decade or so (see for example (Perthame et al. 2014a; Mellet et al. 2017; Degond et al. 2020; David 2023; Chertock et al. 2019; Motsch and Peurichard 2018; David and Perthame 2021)). Taking into account the cells’ migration in the physical growth of the tumor is crucial, especially in order to account for the interactions with the surrounding tissues (competition for space with other healthy and cancerous cells, availability of nutrients etc.). We point out that when $1<m<\infty$, migration is modeled in (1.1) by degenerate diffusion (porous media type equation) which leads to finite speed of propagation of the support of $\rho$ (and thus the support of *n*). The limit $m\rightarrow \infty$ has been at the topic of numerous papers since the original work of Perthame, Quiros and Vazquez (Perthame et al. 2014a) (see for example (Mellet et al. 2017; David and Perthame 2021; Perthame and Vauchelet 2015; Motsch and Peurichard 2018; David 2023) and references therein). The resulting mathematical model is a Hele-Shaw free boundary problem which describes the motion of the interface (the edge of the tumor). We also refer to Friedman (2002) for a review of other free boundary problems appearing in the context of tumor growth modeling.

In Byrne and Drasdo (2009) and Motsch and Peurichard (2018) both agent-based models and continuum models (similar to (1.1) and its singular limit $m\rightarrow \infty$) have been studied numerically. Our model is an intermediate model between these, as it does not track the fate of every individual cell, but nevertheless allows us to take into account the fact that the age distribution in the cell is far from homogeneous. For example, experimental findings have suggested that most mitosis events occur near the edge of the tumor (this is also the case with the individual based models considered in Byrne and Drasdo (2009)) and point to the development of regions of quiescent cells and of necrosis in the core of the tumor. Finally, we point out that (1.2) also shares some similarities with models with phenotypic heterogeneity studied for instance in David (2023), but in that case, each cell is associated to a phenotypic variable $y\in [0,1]$ which affects their proliferation rate but does not change with time.

There can thus be significant advantages in keeping track of both the age and the position of the cells. Even in the simple framework where $\nu$ and *V* are independent of $\theta$, the boundary value problem (1.2) carries more information than (1.1), since the distribution *n* encodes the age structure of the cell population and can thus give critical insight into the model’s prediction. Such a model can then be used to model the effects of various therapies (most therapies target cells that are at some specific stage of their life cycle).

As a final remark, we note that the determination of the parameter $\nu$, $\mu$, *V*, *r* and their dependence on $\theta$ is a delicate issue. We refer for example to Gabriel et al. (2012) for further discussion on this issue.

**Outline of the paper:** The rest of the paper is organized as follows: in Section 2, we state the main result of this paper (the existence of a weak solution for (1.2)). In Section 3, we further discuss the properties of the model and present numerical results to illustrate these. The proof of Theorem 2.1 is developed in Section 4. Additional details about the numerical code are given in Appendix A.

## Main result

A main contribution of this paper is the proof of the existence of a weak solution for the equation (1.2). For simplicity, we take $\mu =0$ in this section (this term does not add any difficulties), so the system reduces to

2.1 $$\begin{aligned} {\left\{ \begin{array}{ll} \partial _{t} n + \partial _{\theta } n - {\textrm{div}\,}_{x} (n \nabla _{x} p) = - \nu (\theta ,p) \, n \qquad & x\in \mathbb {R}^{d},\; \theta>0, \; t>0\\ \displaystyle n(x,0,t) = 2 \int _{0}^{\infty } \nu (\theta ,p)\, n(x,\theta ,t) \, d\theta & x\in \mathbb {R}^{d},\; \; t>0\\ n(x,\theta ,0)= n_{in}(x,\theta ) & x\in \mathbb {R}^{d},\; \theta >0, \end{array}\right. } \end{aligned}$$

where $p=\frac{m}{m-1}\rho ^{m-1}$ with $\rho (x,t) = \int _0^\infty V(\theta ) n(x,\theta ,t)\, dx$. We recall (see (1.7)) that the volume density $\rho (x,t)$ solves

2.2 $$\begin{aligned} \begin{aligned} \partial _t \rho - {\textrm{div}\,}(\rho \nabla p)&= \int _0^\infty V'(\theta ) n(x,\theta ,t)\, d\theta \\&\quad + \int _0^\infty \nu (\theta ,p)(2V(0)-V(\theta )) n(x,\theta ,t)\, d\theta . \end{aligned} \end{aligned}$$

We assume that the coefficients satisfy the following general assumptions: There exist positive constants $V_0, C$ such that

2.3 $$\begin{aligned} V_0\le V(\theta ) \le C,\quad 0\le V'(\theta )\le C, \quad \forall \theta \ge 0,\quad \text{ and } \quad 0\le \nu (\theta ,p) \le C, \quad \forall \theta , \; p\ge 0, \end{aligned}$$

and

2.4 $$\begin{aligned} | V'' (\theta )| , |\partial _{\theta }\nu (\theta ,p)| \le C. \end{aligned}$$

Assumption (2.3) implies in particular that the volume density $\rho (x,t)$ is bounded above and below by the usual number density $\int _0^\infty n(x,\theta ,t)\, d\theta$. Our main result is the following:

### Theorem 2.1

For all $m>2$ and for any initial condition $n_{in}(x,\theta )$ such that

2.5 $$\begin{aligned} n_{in} \in L^1\cap L \log L (\mathbb {R}^d\times (0,\infty )), \quad (|x|^2+\theta ) n_{in} \in L^1 (\mathbb {R}^d\times (0,\infty )), \quad \rho _{in} \in L^\infty (\mathbb {R}^d) \end{aligned}$$

there exists a weak solution $n(x,\theta ,t)$ of (1.2). The function $n(x,\theta ,t)$ is such that

$$ n \in L^\infty (0,T;L^1\cap L \log L (\mathbb {R}^d\times (0,\infty ))), \quad (|x|^2+\theta ) n \in L^\infty (0,T; L^1 (\mathbb {R}^d\times (0,\infty ))) $$

while the density $\rho (x,t)$ and pressure $p(x,t) = \frac{m}{m-1} \rho (x,t)^{m-1}$ are such that

$$ \rho \in L^\infty (0,T; L^1\cap L^\infty (\mathbb {R}^d)), \qquad \nabla p \in L^2(0,T; L^2(\mathbb {R}^d)) $$

and the equation (1.2) is satisfied in the following distributional sense:

2.6 $$\begin{aligned} \begin{aligned}&\int _0^T\int _{\mathbb {R}^d}\! \int _0^\infty n(x,\theta ,t)\\&\big [ -\partial _t \psi (x,\theta ,t)- \partial _{\theta }\psi (x,\theta ,t)+ \nabla _{x} p(x,t) \cdot \nabla _{x}\psi (x,\theta ,t)\\&\qquad + \nu (\theta ,p(x,t))\psi (x,\theta ,t)\big ]\, d\theta \, dx\, dt\\&\quad = \int _{\mathbb {R}^d}\!\int _0^\infty n_{in} (x,\theta ) \psi (x,\theta ,0)\, d\theta \, dx\\&\qquad + \int _0^T \int _{\mathbb {R}^d} \psi (x,0,t) 2 \int _0^\infty \nu (\theta ,p(x,t)) n(x,\theta ,t)\, d\theta \, dx\, dt \end{aligned} \end{aligned}$$

for all $\psi \in \mathcal {D}(\mathbb {R}^d\times [0,\infty )\times [0,T))$. Furthermore, the density $\rho (x,t)$ solves (2.2).

We point out that the term $\int _0^T\!\!\!\int _{\mathbb {R}^d}\! \int _0^\infty n \nabla _{x} p \cdot \nabla _{x}\psi \, d\theta \, dx\, dt$ in the weak formulation (2.6) makes sense if we write it as

$$\begin{aligned} \int _0^T\!\!\!\int _{\mathbb {R}^d}\left( \int _0^\infty n \nabla _{x}\psi \, d\theta \right) \cdot \nabla _{x} p \, dx\, dt \end{aligned}$$

since $$ |\int _0^\infty n \nabla _{x}\psi \, d\theta |\le C\Vert \nabla \psi \Vert _{L^\infty } \rho \in L^2(0,T;L^2(\mathbb {R}^d))$$ and $$\nabla _{x} p \in L^2(0,T;L^2(\mathbb {R}^d))$$.

The restriction $m>2$ might not seem natural when considering the volume density equation (2.2) since the divergence term can be written (as is classical with the porous media equation) in the form $\Delta \rho ^m$ and corresponds to degenerate diffusion for all $m> 1$ (and linear diffusion when $m=1$). On the other hand, this restriction is classical for cross-diffusion equations (David 2023), even in the case of just two cross-diffusive populations (Di Francesco et al. 2018). This condition will be essential to proving the convergence of the gradient pressure in our proof. Note that the divergence term in (2.1) can also be written as $m\, {\textrm{div}\,}(n \rho ^{m-2} \nabla \rho )$, and is singular when $m<2$.

Under assumption (2.3), the density equation (2.2) implies in particular

$$\begin{aligned} \partial _t \rho - {\textrm{div}\,}(\rho \nabla p) \le C\rho , \qquad p=\frac{m}{m-1} \rho ^{m-1}. \end{aligned}$$

And multiplying this equation by $m\rho ^{m-2}$, we deduce the following equation for the pressure *p*:

2.7 $$\begin{aligned} \partial _t p - (m-1) p \Delta p - |\nabla p|^2 \le (m-1) C p . \end{aligned}$$

This equation will play a role in the proof and it implies the following classical result:

### Proposition 2.2

(Finite speed of propagation of the support) Under the assumptions of Theorem 2.1, assume further that there exists $R_0$ such that $n_{in }(x,\theta ) =0$ for all $|x|\ge R_0$ and $\theta \ge 0$. Then

$$n(x,\theta ,t) = 0 \quad \text{ for } \text{ all } |x|\ge {\overline{R}} + e^{(m-1)Ct} \text{ and } \text{ for } \text{ all } \theta \ge 0$$

for some ${\overline{R}}$ depending on $R_0$ and $\Vert \rho _{in}\Vert _{L^\infty }$.

### Proof

Setting $A:= (m-1)C$, we note that given a unit vector *e*, the function ${\overline{p}} (x,t) = e^{At}\left( e^{At}-\sqrt{A} (x\cdot e -{\overline{R}}) \right) _+$ is a super-solution for (2.7) with support in $\{ x\cdot e \ge {\overline{R}} + \frac{1}{\sqrt{A}} e^{At}\}$ and satisfying ${\overline{p}}(x,0) \ge 1+\sqrt{A} ({\overline{R}}-R_0)$ on $\textrm{Supp}\,p_{in}$. Taking ${\overline{R}}$ large enough so that ${\overline{p}}(x,0) \ge p_{in}(x)$, the result follows from the comparison principle applied to (2.7). $\square$

On the other hand, it is not obvious that we can get a bound on *R*(*t*) that is uniform with respect to *m*, the main obstacle being the term $\int _0^\infty V'(\theta ) n (\theta )\, d\theta$. This term is responsible for another significant difference with (1.1): even if we assume that $\nu (\theta ,p)=0$ for all $p\ge p_M$ and that $p_{in}(x)\le p_M$ it is not true in general that $p(t,x)\le p_M$ for all $t\ge 0$.

Finally, we note that the proof of Theorem 2.1 could easily be adapted to the pressure limited growth model (1.4). In fact, this model has slightly better properties since if we assume that $r(p)=0$ for $p\ge p_M$ (i.e. preventing not only mitosis, but also the growth of cells when $p\ge p_M$), then $p(x,t)\le p_M$ for all $t\ge 0$ if it is true at $t=0$. This follows from the fact that the pressure solves

$$ \partial _t p - (m-1) p \Delta p - |\nabla p|^2 \le (m-1) C r(p) p $$

and the usual maximum principle.

Before we give the proof of Theorem 2.1, we present in the next section some numerical results which illustrate other important properties of our model.

## Properties of the model and numerical results

When the volume $V(\theta )$ and proliferation rate $\beta (\theta )$ are independent of $\theta$, the volume density $\rho (t,x) = V_0\int _0^\infty n(t,x,\theta )\, d\theta$ solves the classical porous media type equation with growth term (1.6). This classical model has been extensively studied both theoretically and numerically (Friedman 1983; Vazquez 2006) (including the singular limit $m\rightarrow \infty$, see Perthame et al. (2014a); Mellet et al. (2017)). Classical properties include finite speed expansion of the support and the existence of traveling wave solutions which describe the asymptotic spreading speed of any compactly supported initial configuration. Similar properties are expected to hold for general $V(\theta )$ and $\beta (\theta )$ and will be investigated numerically below. But we also note that even in the simplest case mentioned above, our model provides additional information concerning the spreading of tumors under mechanical process described in the introduction. In this section, we further discuss some properties of the model and present numerical results to illustrate them.

### Numerical setting

Throughout this section, we fix $m=4$ (so $p = \frac{4}{3}\rho ^3$) and we take

$$ \nu (\theta ,p)=\beta (\theta ) \left( \frac{4}{3} -p\right) _+, $$

where (following Gabriel et al. (2012))

3.1 $$\begin{aligned} \beta (\theta )={\left\{ \begin{array}{ll} 0 & \text{ if } \theta \le \theta _0 \\ \frac{(\theta -\theta _0)^2}{\sigma (2\sigma ^2+2\sigma (\theta -\theta _0) + (\theta -\theta _0)^2)}& \text{ if } \theta \ge \theta _0, \end{array}\right. } \end{aligned}$$

with $\theta _0=5$ and $\sigma = 1$. In Sections 3.2-3.3 we restrict ourselves to Equation (1.2) with $V(\theta )\equiv V_0=1$. We will consider (1.4) and changing volumes in Section 3.4.

For computation purposes, we need to take the age variable $\theta$ in a bounded interval $[0,\theta _{max}]$. When we can take $\theta _{max}$ larger than the maximum computation time, this maximum age does not play a role in determining the solution. But since cells do not live forever, it can be relevant (and numerically less costly) to take $\theta _{max}\le T$. In that case, one needs to specify what happens to cells when they reach the age $\theta =\theta _{max}$. In the numerical computations that we present below, we assume that these “older" cells are still physically present (they contribute to the pressure), but they are no longer proliferating. This amounts to assuming that $\beta (\theta ) =0$ when $\theta >\theta _{max}$ (inactive cells).

Note that it would be easy to assume instead that these cells are proliferating with a constant rate ($\beta (\theta )=\beta (\theta _{max})$ for $\theta >\theta _{max}$). In practice, we actually see very little difference between these two settings in the simulations: indeed, older cells are primarily found in the center of the tumor, where the proliferation is already negligible because of the high pressure $p\sim 4/3$. In addition, we will see that when the death/removale of cells is taken into account ($\mu \ne 0$), the number of cells of age $\theta$ converges to zero exponentially fast and taking $\theta _{max}$ large enough will again lead qualitatively to the same results as when $\theta _{max}=+\infty$.

Below, we present and discuss 2D simulations where the equation is set in the square $[-10,10]\times [-10,10]\subset \mathbb {R}^2$ and the system is supplemented with Neumann boundary conditions. Additional information about the numerical scheme can be found in Appendix A.

**Fig. 1:**
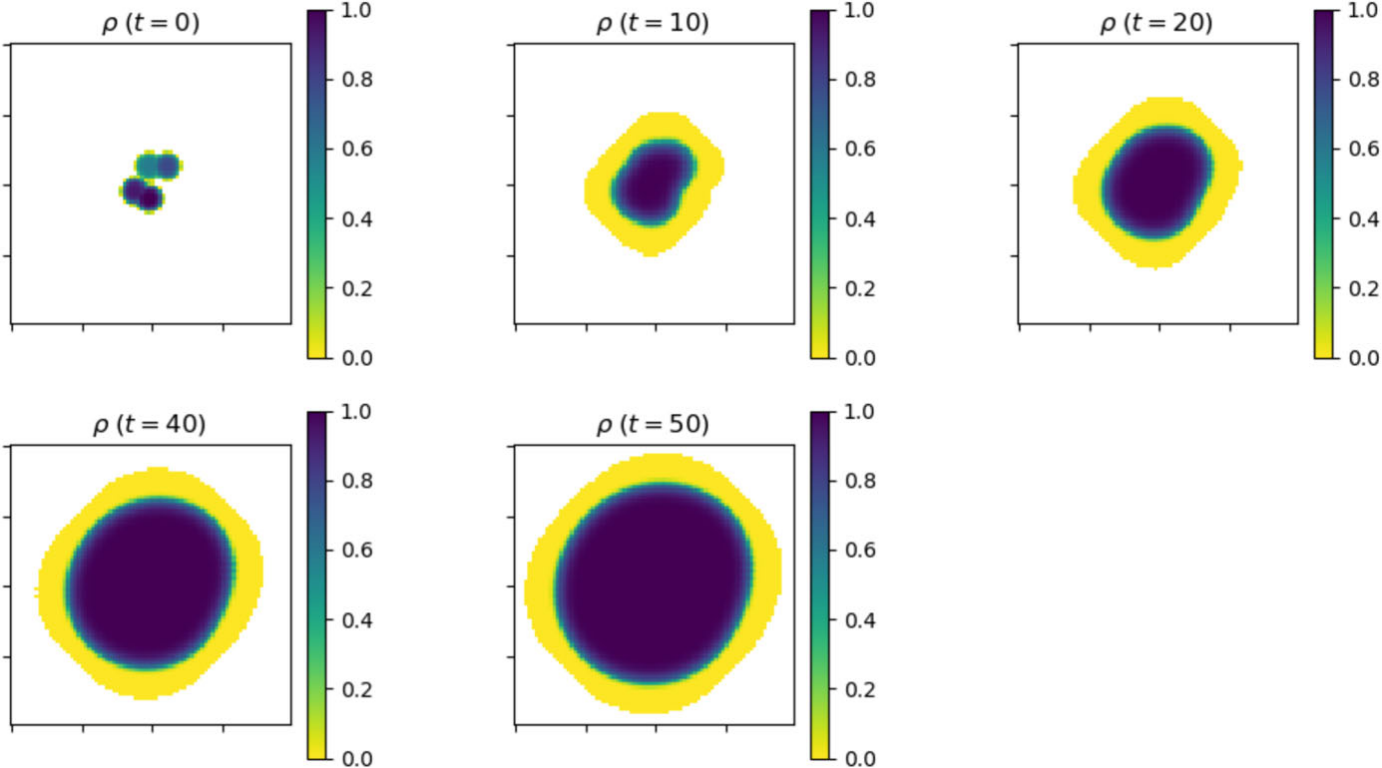
The density $x\mapsto \rho (x,t)$ for a 2-dimensional tumor at time $t=0$, 10, 20, 40 and 50. Although the initial data (top left plot) is not radially symmetric, the tumor shape visually approaches a disk as *t* increases

### Growth of the tumor and mitosis events

Figure 1 shows the evolution of the density $\rho (x,t) = V_0\int _0^\infty n(x,\theta ,t)\, d\theta$ with $\theta _{\max } =20$ for some non-symmetric initial data. We note that despite the lack of symmetry of the initial data, the tumor appears to converge to a disk as *t* increases. This behavior is consistent with the large time asymptotic of the solutions of the porous media equation (Vazquez 2006). With radially symmetric initial data, the tumor remains symmetric for all positive time. One of the most interesting feature of our model, compared with macroscopic models that only describe the evolution of the density $\rho (x,t)$, is that it keeps track of where proliferation is taking place. Indeed, in vitro experiments with cancerous cells suggest that the growth of the tumor is mostly due to the proliferation of cells in a small region near, but not only at, the outer edge of the tumor (cell proliferation in some small region near the edge also plays a fundamental role in the expansion of bacteria colonies (Aawaz et al. 2024; Julian and Wimpenny 1979)).

**Fig. 2:**
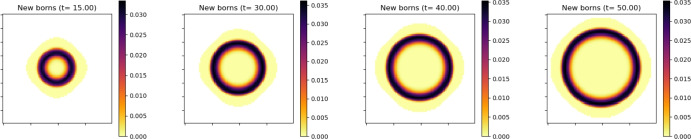
This figure shows the number of mitosis events inside the tumor at various stage of the tumor growth. The highest concentration of mitosis events can be found in an annulus which has constant width and stays at some positive distance from the edge of the tumor

Figure 2 shows the distribution of cells that just experienced mitosis $n(x,\theta =0,t)$ and clearly shows that the model is consistent with this observation: most mitosis events take place in an annulus close to the edge of the support of *n*. The width of the annulus and its distance to the edge do not change with time.

**Fig. 3:**
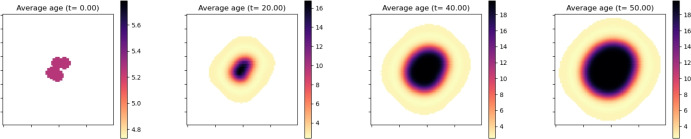
Average age $\Theta (x,t)$ of the cells across the tumor at time $t=0,\, 20,\, 40$ and 50. The lowest average age (light yellow) is found in a ring that has constant width and stays at some positive distance from the edge of the tumor

This phenomena is related to another feature identified in experiments, the notion of “surfing cells" at the front (Farrell et al. 2017). This corresponds to the fact that cells at the outer edge of the tumor are being pushed by the growing bulk density and are thus not primarily new born cells. This is illustrated in Figure 3 which represents the average age

3.2 $$\begin{aligned} \Theta (x,t):=\frac{\int _0^\infty \theta n(x,\theta ,t)\, d\theta }{\int _0^\infty n(x,\theta ,t)\, d\theta } , \end{aligned}$$

as a function of *x* and at various time. It shows that this average age is smallest in a ring away from the outer edge.

**Fig. 4:**
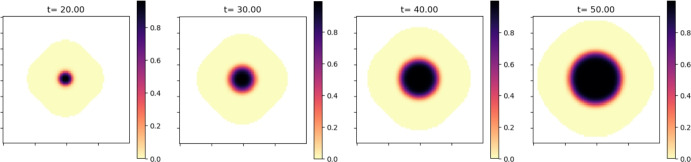
The spatial distribution of the cells of the maximum numerical age $\theta _{max}=20$ shows an accumulation of old cells in the core of the tumor (necrotic core)

For comparison, Figure 4 shows the distribution of cells of age $\theta _{max}$ across the tumor. These are the cells that have reached their maximal life span and they can be found mostly in the center of the tumor (we can also interpret this as a necrotic core).

### Long time behavior and traveling wave solutions.

We now turn our attention to the behavior of the solutions for large time. Two important features of tumor growth are the speed of spreading of the tumor and the asymptotic age distribution of the cells across the tumor. Indeed, a motivation for the study of age-structured models is the development (or improvement) of therapies. Since cells in different phases of their life cycle are affected differently by therapies, a precise description of the age distribution of the tumor cells can help develop effective strategies to limit their growth.

**Space homogeneous model.** There is an extensive literature (see for example (Dyson et al. 2002; Mischler and Scher 2016; Gurtin and MacCamy 1974, 1979)) devoted to space-homogeneous age-structured models such as (1.10). The long time asymptotic is a classical problem. Indeed, the system (1.10) has particular solutions of the form

3.3 $$\begin{aligned} n_\infty (\theta ,t) = c e^{\lambda t} \varphi (\theta ), \end{aligned}$$

where $$\varphi $$ solves

$$ \lambda \varphi + \varphi ' = - \beta (\theta ) \, \varphi - \mu (\theta ) \varphi , \qquad \varphi (0)=1, $$

and $\lambda$ is determined by the condition

$$ 2 \int _0^\infty \beta (\theta )\, \varphi (\theta ) \, d\theta = 1. $$

This parameter $\lambda$ thus characterizes the *exponential growth* of the cell population, while the function $\varphi$ describes the asymptotic age distribution of the cells. In particular, it can be proved (see Dyson et al. (2002)) that for any nonzero initial data, the solution of (1.10) satisfies:

$$ \frac{ n(\theta ,t)}{\int _0^\infty n(\theta ',t) d\theta ' }\rightarrow \varphi (\theta )\qquad \text{ as } t\rightarrow + \infty . $$

**Space-dependent model: traveling wave solutions.** When pressure and space variable are taken into account, the asymptotic behavior of the solution is very different. An important property of the reaction-diffusion equation (1.1) is the existence of traveling wave solutions and the finite speed of spreading (see for example (Perthame and Vauchelet 2015; Perthame et al. 2014b) and references therein for traveling wave solutions in the context of tumor growth). In particular, we recall that when $F(p)=p_M-p$, (1.1) admits solutions of the form $\bar{u}(x-ct)$ for all $c\ge c^*$ where the smallest speed $c^*$ is also the spreading speed of compactly supported solutions.

The existence of traveling wave solutions for the simple age-structured model

**Fig. 5:**
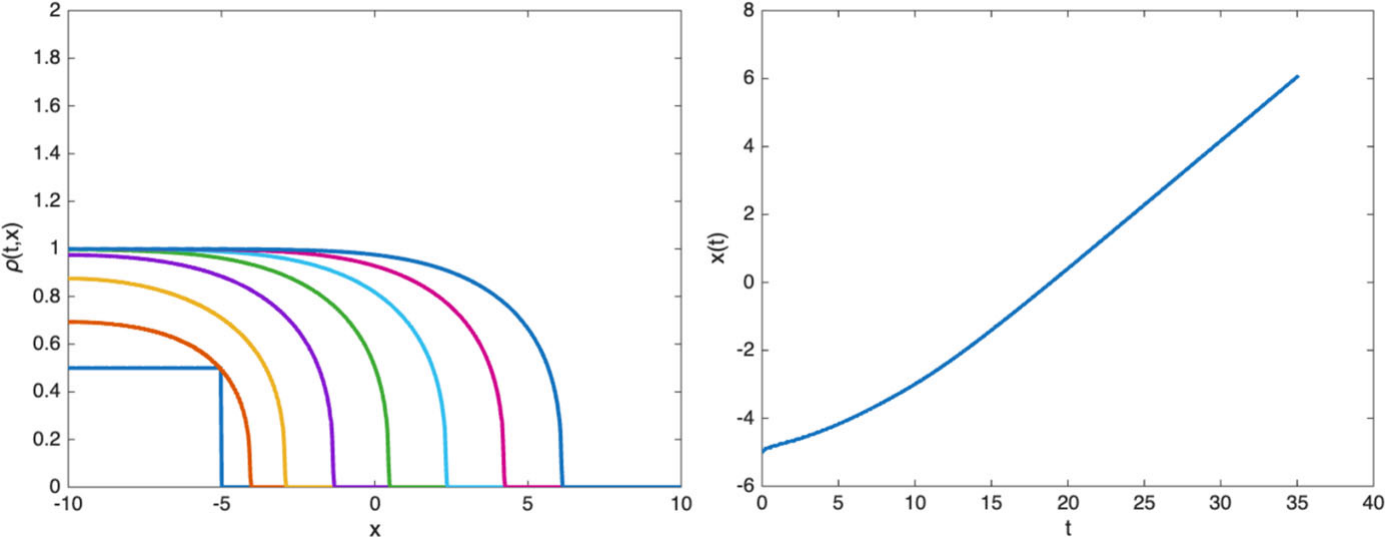
Development of a front propagating with constant speed. The left figure shows the evolution of the one-dimensional density $\rho (x,t)$ starting from a characteristic function $\frac{1}{2} \chi _{x\le -5}$ at time $t=0$, 5, 10, 15, 20, 25, 30 and 35. The right figure shows the position of the front *x*(*t*) as a function of time (defined as $\rho (x(t),t)=1/2$). As expected from traveling waves, the front motion is asymptotically linear in time

3.4 $$\begin{aligned} {\left\{ \begin{array}{ll} \partial _t n + \partial _{\theta } n - {\textrm{div}\,}_{x} (n \nabla _{x} p) = - \nu (\theta ,p) \, n \\ \displaystyle n(x,0,t) = 2 \int _{0}^{\infty } \nu (\theta ,p)\, n(x,\theta ,t) \, d\theta , \end{array}\right. } \end{aligned}$$

with $\nu (\theta ,p) = \beta (\theta )(p_M-p)_+$ can be observed numerically in one dimension as shown in Figure 5:

After some initial transition time, the solution is asymptotically close to a traveling wave, which is a solution of (3.4) of the form $n(x,\theta ,t) = \bar{n} (x-ct,\theta )$ where the corresponding pressure variable $\bar{p}(x)$ satisfies the boundary condition

3.5 $$\begin{aligned} \lim _{x\rightarrow -\infty } \bar{p}(x) = p_M, \quad \lim _{x\rightarrow +\infty } \bar{p}(x) = 0 . \end{aligned}$$

A rigorous mathematical justification of this fact is the object of a forthcoming work.

**Fig. 6:**
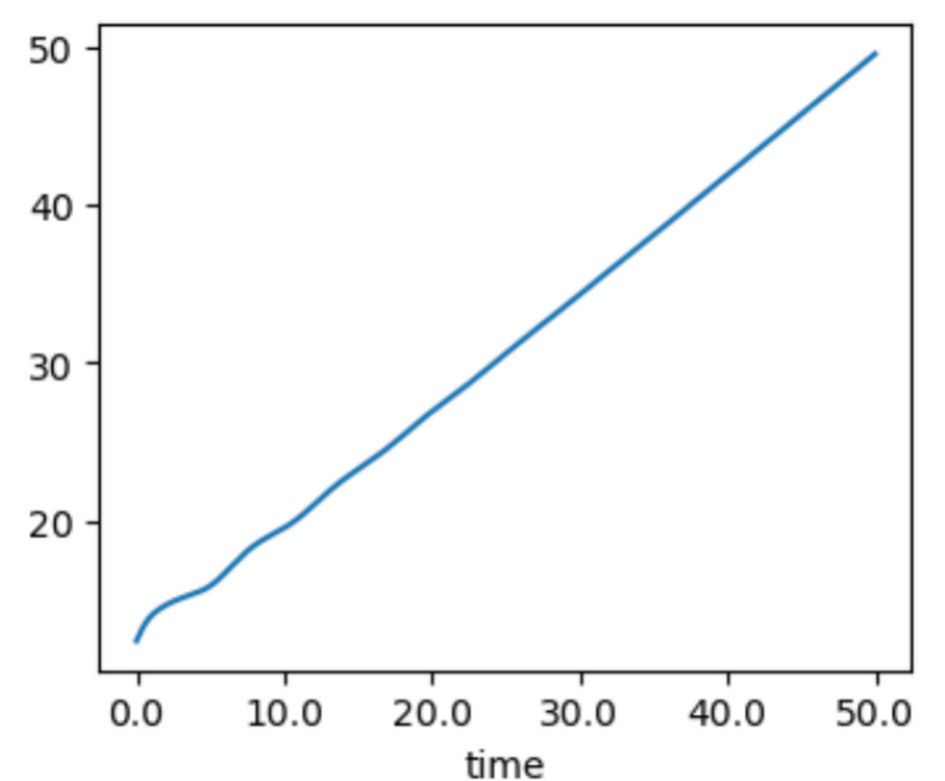
Growth of $\left( \int _\Omega \rho (x,t) \; dx\right) ^{1/2}$ over time for a two dimensional tumor. The asymptotic linear growth is consistent with the linear growth of the diameter of the tumor and with the existence of traveling wave solutions

This implies that the growth of the tumor is no longer exponential and that the speed of the traveling wave characterizes the growth of the diameter of the tumor. In two dimension, for example, this implies that the total mass of the tumor grows quadratically, a phenomenon that can be confirmed numerically (see Figure 6).

Focusing on the behavior of $n(x,\theta ,t)$ for large *t* we see numerically that the traveling wave solution satisfies

3.6 $$\begin{aligned} n(x,\theta ,t) = \bar{n}(x-ct,\theta ) \sim \phi (x+c(\theta -t))\quad \text{ as } t\rightarrow \infty , \end{aligned}$$

for fixed *x* and $\theta$ and for some profile $\phi$. This observation is consistent with the equation since (3.4) implies $\partial _t n +\partial _{\theta } n=-c\partial _x \bar{n}+ \partial _{\theta }\bar{n} \rightarrow 0$ as $x\rightarrow -\infty$. This can be explained as follows: as the tumor is spreading, the pressure at a given *x* converges to $p_M$ when $t\rightarrow \infty$ so that both diffusion and proliferation become negligible. The asymptotic behavior (3.6) simply captures the aging of the cells.

**Death rate: expansion vs. regression of the tumor.** The long time behavior of the solutions is quite different when we include the death rate $\mu (\theta )$ in the model:

3.7 $$\begin{aligned} {\left\{ \begin{array}{ll} \partial _t n + \partial _{\theta } n - {\textrm{div}\,}_{x} (n \nabla _{x} p) = - \nu (\theta ,p) \, n -\mu (\theta )n \\ \displaystyle n(x,0,t) = 2 \int _0^\infty \nu (\theta ,p)\, n(x,\theta ,t) \, d\theta . \end{array}\right. } \end{aligned}$$

**Fig. 7:**
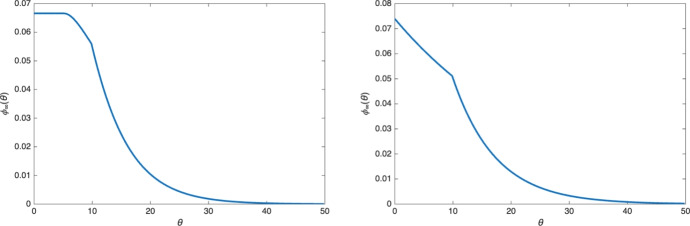
The function $\phi _\infty (\theta )$ when $\mu (\theta )$ is given by (3.8) and $\beta (\theta )$ is given by (3.1) (left) or $\beta (\theta ) \equiv 1$ (right). In both cases, the discontinuity of $\phi _\infty '$ at $\theta =10$ is due to the discontinuity of the death rate $\mu (\theta )$. In the left plot, both the death rate $\mu (\theta )$ and the proliferation rate $\beta (\theta )$ vanish for $\theta \in [0,5]$ resulting in $\phi _\infty$ being constant in that range

First, we observe numerically that for large enough $\mu$, the tumor might shrink and eventually disappear ($\lim _{t\rightarrow \infty } n(t) =0$), while for small $\mu$, a moving front with positive speed develops as in the case $\mu =0$. We also note that traveling wave solutions cannot satisfy (3.5) when $\mu >0$ since the right-hand side of (3.7) no longer vanishes for $p=p_M$. Finally, when the tumor spreads, numerical simulations show that there exists a unique profile $\phi _\infty (\theta )$ such that

$$\begin{aligned} n(x,\theta ,t) \rightarrow \phi _\infty (\theta ) \quad \text{ as } t\rightarrow \infty , \end{aligned}$$

for all $x\in \mathbb {R}^d$ (which is very different from the asymptotic behavior (3.6)). Examples of this profile $\phi _\infty (\theta )$ are shown in Figure 7 when $\mu$ is given by

3.8 $$\begin{aligned} \mu (\theta )= {\left\{ \begin{array}{ll} 0 & \text{ if } \theta < 10 ,\\ 0.1 & \text{ if } \theta \ge 10 , \end{array}\right. } \end{aligned}$$

and for two different choices of $\beta (\theta )$.

This behavior can be investigated analytically: going back to (3.4) we see that the asymptotic profile $\phi _\infty (\theta )$ (if it exists) must solve

3.9 $$\begin{aligned} {\left\{ \begin{array}{ll} \phi '(\theta ) = -\nu (\theta ,p_0) \phi (\theta ) - \mu (\theta )\phi (\theta ), \qquad \theta \in (0,\infty ),\\ \displaystyle \phi (0)= 2 \int _0^\infty \nu (\theta ,p_0) \phi (\theta )\, d\theta , \\ \displaystyle p_0 = \frac{m}{m-1}\left( \int _0^\infty V_0 \phi (\theta ) \, d\theta \right) ^{m-1}. \end{array}\right. } \end{aligned}$$

This is a particular case of the growth fragmentation model considered for instance in Gurtin and MacCamy (1974). Using the monotonicity of $p\mapsto \nu (p,\theta )$, we can show the following result:

#### Proposition 3.1

Assume that $m>1$, that $\mu (\theta )$ satisfies $\int _0^\infty \mu (\theta )\, d\theta =\infty$ (every cell dies eventually) and that $\nu (\theta ,p)$ satisfies (1.3). There exist unique $p_0\in (0,p_M)$ and $\phi _\infty (\theta )$ solution of (3.9) if and only if

3.10 $$\begin{aligned} \int _0^\infty \mu (\theta ) e^{-\int _0^\theta \nu (s,0) +\mu (s)\, ds}\, d\theta < \frac{1}{2}. \end{aligned}$$

#### Proof

For a given $p_0$ the solutions of the first equation in (3.9) are given by

$$\begin{aligned} \phi (\theta ) = \alpha e^{-\int _0^\theta \nu (s,p_0) +\mu (s)\, ds} \end{aligned}$$

with $\alpha \in \mathbb {R}$. The second equation in (3.9) is then equivalent to

3.11 $$\begin{aligned} 1 = 2 \int _0^\infty \nu (\theta ,p_0) e^{-\int _0^\theta \nu (s,p_0) +\mu (s)\, ds}\, d\theta \end{aligned}$$

which is a condition on $p_0$. The non-degeneracy condition $\int _0^\infty \mu (\theta )\, d\theta =\infty$ implies

$$ \int _0^\infty \nu (\theta ,p_0) e^{-\int _0^\theta \nu (s,p_0) +\mu (s)\, ds}\, d\theta = 1 -\int _0^\infty \mu (\theta ) e^{-\int _0^\theta \nu (s,p_0) +\mu (s)\, ds}\, d\theta $$

and so (3.11) is equivalent to

$$ \int _0^\infty \mu (\theta ) e^{-\int _0^\theta \nu (s,p_0) +\mu (s)\, ds}\, d\theta = \frac{1}{2}. $$

Under assumptions (1.3), the left-hand side is monotone increasing with respect to $p_0$ and equal to 1 when $p_0=p_M$. The existence of a unique $p_0\in (0,p_M)$ satisfying (3.11) is thus equivalent to the condition (3.10).

Under condition (3.10), the discussion above shows that there is a unique $p_0\in (0,p_M)$ such that (3.11) holds. We deduce the existence of $\phi _\infty$ solution of (3.9) given by

$$\begin{aligned} \phi _\infty (\theta ) = \alpha e^{-\int _0^\theta \nu (s,p_0) +\mu (s)\, ds} \end{aligned}$$

where the constant $\alpha$ is determined by the last equation in (3.9). $\square$

In view of this proposition, we conjecture that (3.10) identifies the threshold that characterizes the long time behavior (expansion vs. regression) of the tumor mass and that when (3.10) holds, the long time dynamic of the tumor will be described by traveling wave solutions of (3.4) which satisfy the boundary conditions

$$ \lim _{x\rightarrow -\infty } \bar{p}(x) = p_0, \quad \lim _{x\rightarrow -\infty } \bar{n}(x,\theta ) = \phi _\infty (\theta ), \qquad \lim _{x\rightarrow +\infty } \bar{p} (x)=\lim _{x\rightarrow +\infty } \bar{n}(x,\theta ) = 0. $$

This conjecture is easy to verify when $\mu$ and $\nu (p)=\beta (p_M-p)_+$ are independent of $\theta$. In that case, condition (3.10) is equivalent to $\mu <\beta \, p_M$ and the equation for the density reduces to

$$\begin{aligned} \partial _t\rho - \Delta \rho ^m = \rho (\beta (p_M-p)_+ -\mu ). \end{aligned}$$

So the condition $\mu <\beta \, p_M$ is indeed a necessary and sufficient condition for this equation to have a positive stable stationary solution.

When $\mu$ is independent of $\theta$ and $\nu (p,\theta )=\beta (\theta ) (p_M-p)_+$ is a function of $\theta$, we can test this conjecture numerically: we define $\mu _c$ as the value of $\mu$ for which we have equality in (3.10), that is

$$ \int _0^\infty \mu _c e^{-\int _0^\theta \beta (s) p_M +\mu _c\, ds}\, d\theta = \frac{1}{2}. $$

When $\beta (\theta )$ is given by (3.1), this critical value can be determined numerically and is given by $\mu _c=0.0928$.

**Fig. 8:**
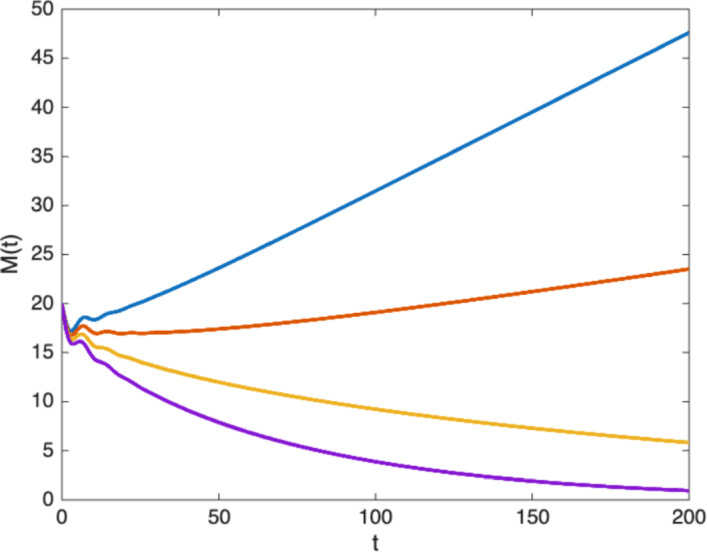
Evolution of the mass of the tumor, $M(t)=\int _{\Omega }\rho (x,t)dx$, when $\beta (\theta )$ is given by (3.1) and for different values of the death rate $\mu$ both below and above the threshold $\mu _c=0.0928$ (determined by condition (3.10)). For $\mu =.08$ (blue curve) and $\mu =0.09$ (red curve), the tumor is expanding in time, while for $\mu =0.1$ (yellow curve) and $\mu =0.11$ (purple curve), the tumor is regressing

Figure 8 shows the evolution of the mass of the tumor for different values of $\mu$ both above and below $\mu _c$. The behavior is consistent with condition (3.10) and show that the tumor is expanding when $\mu <\mu _c$ and shrinking when $\mu >\mu _c$.

### Pressure-limited growth and non-constant volume

The discussion has so far focused on the pressure-limited proliferation model (1.2) with constant volume $V(\theta )\equiv 1$. We will now compare it to the pressure-limited aging model (1.4) with age dependent volume $V(\theta )$. We assume that the volume of a cell grows linearly until it has doubled in size when $\theta =\theta _0=5$:

3.12 $$\begin{aligned} V(\theta ) = {\left\{ \begin{array}{ll} V_0\left( 1 + \frac{\theta }{\theta _0}\right) & \text{ if } \theta \le \theta _0 \\ 2V_0 & \text{ if } \theta \ge \theta _0. \end{array}\right. } \end{aligned}$$

Cells of age $\theta >\theta _0$ will then split, with probability $\nu (p,\theta )$, into two daughter cells of volume $V_0$. Since this splitting is now a volume preserving process we assume that it is independent of the pressure *p*: We take

$$\begin{aligned} \nu (\theta ,p)=\beta (\theta ), \qquad \text{ with } \beta (\theta )=0 ,\; \text{ for } \text{ all } \theta \le \theta _0. \end{aligned}$$

We obtain the following model:

3.13 $$\begin{aligned} {\left\{ \begin{array}{ll} \partial _t n + r(p) \partial _{\theta } n - {\textrm{div}\,}_{x} (n \nabla p) = - r(p) \beta (\theta ) n - \mu (\theta ) n \qquad & x\in \mathbb {R}^d,\; \theta>0, \; t>0\\ \displaystyle n(x,0,t) = 2 \int _0^\infty \beta (\theta ) n(x,\theta ,t) \, d\theta & x\in \mathbb {R}^d,\; \; t>0\\ n(x,\theta ,0)= n_{in}(x,\theta ) & x\in \mathbb {R}^d,\; \theta >0, \end{array}\right. } \end{aligned}$$

with $p(x,t)= \frac{m}{m-1} \left( \int _0^\infty V(\theta )n(x,\theta ,t)\, d\theta \right) ^{m-1}$. Crucially, the cell’s growth (or aging) is now limited by pressure: we take

$$\begin{aligned} r(p) = (p_M-p)_+ . \end{aligned}$$

In the numerical simulations presented in this section, we compare the evolution of a tumor for the two models, referred to as Model (I) and Model (II) as follows:

(I) The pressure-limited proliferation model (1.2) that we have considered previously: in this model the pressure *p* appears in the proliferation frequency $\nu (\theta ,p)= \beta (\theta )\left( p_M - p\right) _+$ and the cells’ volume is constant $V(\theta )=V_0=1$ (cells duplicate into two daughter cells of the same size).
(II) The pressure-limited aging model (3.13) with age dependent volume $V(\theta )$ given by (3.12) (with $V_0=1$ as well) and $r(p) = (p_M-p)_+.$

In both models, the proliferation rate $\beta (\theta )$ is given by (3.1), the death rate $\mu (\theta )$ is given by (3.8) and $m=4$ (so $p=\frac{4}{3} \rho ^3$).

Before presenting some numerical results, we need to make some important remarks concerning the long time behavior of the solutions of Model (II): if the solution of (3.13) converges to a space-homogeneous age profile $\phi _\infty (\theta )$ as *t* approaches $\infty$, then $\phi _\infty (\theta )$ solves

3.14 $$\begin{aligned} {\left\{ \begin{array}{ll} r(p_0) \phi '(\theta ) = - r(p_0) \beta (\theta ) \phi (\theta ) -\mu (\theta ) \phi (\theta ), & \theta \in (0,+\infty )\\ \displaystyle \phi (0)= 2 \int _0^\infty \beta (\theta ) \phi (\theta )\, d\theta \\ \displaystyle p_0 = \frac{m}{m-1}\left( \int _0^\infty V(\theta ) \phi (\theta ) \, d\theta \right) ^{m-1} \in [0,p_M]. \end{array}\right. } \end{aligned}$$

Compared with (3.9), we notice that a new phenomena emerges: When $p_0=p_M$, the first equation reduces to $\mu (\theta ) \phi (\theta )=0$ (since $r(p_M)=0$). In particular if the set $S=\{ \theta ;\, \mu (\theta )=0\}$ is not empty, then any function $\phi (\theta )$ supported on *S* with pressure $p=p_M$ is a stationary solution. In that case the long time asymptotic age distribution is not uniquely determined by the PDE and might depend on the initial data.

**Fig. 9:**
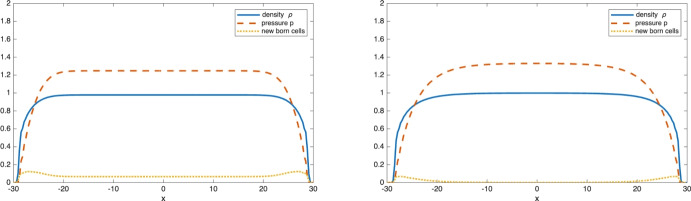
The density $\rho (x,T)$, pressure *p*(*x*, *T*) and distribution of newborn cells at time $T=80$. For Model (I) - pressure-limited proliferation - on the left and Model (II) - pressure limited growth - on the right

This phenomena can be observed numerically when $\mu$ is given by (3.8) (for which we have $S=[0,10]$). Figure 9 shows the volume density $\rho (x,T)$, pressure *p*(*x*, *T*) and distribution of newborn cells at time $T=80$, for a tumor that grew from a compactly supported initial condition supported in the interval $[-2,2]$. With Model (II) (right plot) there is almost no mitosis taking place in the core of the tumor: as aging cells grow in size they increase the pressure in their surroundings. This increased pressure slows down the aging process which eventually stops when *p*(*t*, *x*) reaches the homeostatic pressure $p_M$. In contrast, Model (I) (left plot) allows cells to age and eventually die even when the pressure is high. The dying cells leave room for further proliferation and the rate of mitosis is strictly positive even in the center of the tumor.

**Fig. 10:**
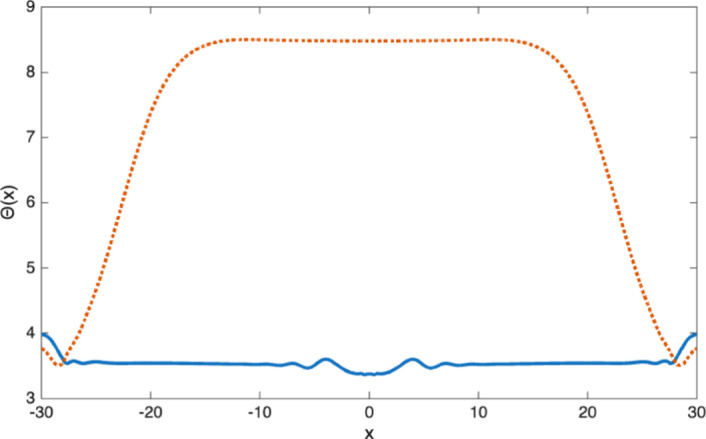
Comparison of the average age distribution inside the tumor at $T=80$ for Model (I) (dashed line) and Model (II) (solid line)

Both the interrupted aging process and non-uniqueness of the age distribution inside the tumor are also evident in Figure 10 which displays the average age $\Theta (x,T)$ (see (3.2) for the definition) at time $t=T$ and throughout the tumor. While $\Theta (x,T)$ is constant in the core of the tumor for Model (I) (and equal to the average age of the asymptotic distribution $\phi _\infty (\theta )$ of Figure 7), it is much smaller and not constant for Model (II), indicating that the cell age distribution $\theta \rightarrow n(x,\theta ,T)$ is not space homogeneous inside the tumor. Since very few cells reach the dying age $\theta =10$ in these simulations, we observe no significant differences between this case and the case $\mu =0$.

Next, we look for stationary solutions of (3.14) with pressure $p_0<p_M$. We have the following result (which is the counterpart of Proposition 3.1 for Model (II)):

#### Proposition 3.2

There exists a unique $p_0\in (0,p_M)$ and $\phi _\infty (\theta )$ solution of (3.14) if and only if

3.15 $$\begin{aligned} \int _0^\infty \beta (\theta ) \exp \left( -\int _0^\theta \beta (s) + \frac{\mu (s)}{r(0)}\, ds\right) d\theta > \frac{1}{2}, \end{aligned}$$

and

3.16 $$\begin{aligned} \lim _{p\rightarrow p_M} \int _0^\infty \beta (\theta ) \exp \left( -\int _0^\theta \beta (s) + \frac{\mu (s)}{r(p)}\, ds\right) d\theta <\frac{1}{2}. \end{aligned}$$

#### Proof

We proceed as in the proof of Proposition 3.1 to show that a stationary solution $\phi _\infty (\theta )$ with $p_0\in (0,p_M)$ exists if and only if

$$ \int _0^\infty \beta (\theta ) \exp \left( -\int _0^\theta \beta (s) + \frac{\mu (s)}{r(p_0)}\, ds\right) d\theta = \frac{1}{2}. $$

Since the quantity in the left hand side is monotone decreasing with respect to $p_0$, the intermediate value theorem determines a unique value $p_0\in (0,p_M)$ provided conditions (3.15) and (3.16) are satisfied. The profile is then given by

$$ \phi _\infty (\theta ) = \alpha \exp \left( -\int _0^\theta \beta (s) +\frac{\mu (s)}{r(p_0)}\, ds\right) $$

where the constant $\alpha$ is determined by the last equation in (3.14). $\square$

When condition (3.15) fails (which happens when $\mu$ is too large), then $\phi \equiv 0$ is the only solution of (3.14) with $p_0<p_M$ and the tumor is expected to shrink and disappear as $t\rightarrow \infty$.

The second condition (3.16) is trivially satisfied when the death rate $\mu (\theta )$ is positive for all $\theta$ (so $S=\emptyset$). Otherwise, we note that when $S=[0,\theta _0]$, as is the case with (3.8), we find (recall that $r(p_M)=0$)

$$\begin{aligned} \lim _{p\rightarrow p_M}&\int _0^\infty \beta (\theta ) \exp \left( -\int _0^\theta \beta (s) + \frac{\mu (s)}{r(p)}\, ds\right) d\theta \\&\quad = \int _0^{\theta _0} \beta (\theta ) \exp \left( -\int _0^\theta \beta (s)\, ds\right) d\theta \\&\quad = 1-\exp \left( -\int _0^{\theta _0} \beta (s)\, ds \right) \end{aligned}$$

so condition (3.16) is equivalent to $\exp \left( -\int _0^{\theta _0} \beta (s)\, ds \right) >\frac{1}{2}.$ When this condition fails, the solution $n(x,\theta ,t)$ of (1.4) will saturate to the maximum pressure $p=p_M$ with an age distribution supported in $S=\{ \mu (\theta )=0\}$ that is not uniquely determined by the PDE but depends on the initial condition. This is the case in particular when $\mu$ is given by (3.8) (that is $S=[0,10]$) and $\beta (\theta )$ is given by (3.1), which is consistent with the behavior observed in Figures 9 and 10 and discussed above.

**Fig. 11:**
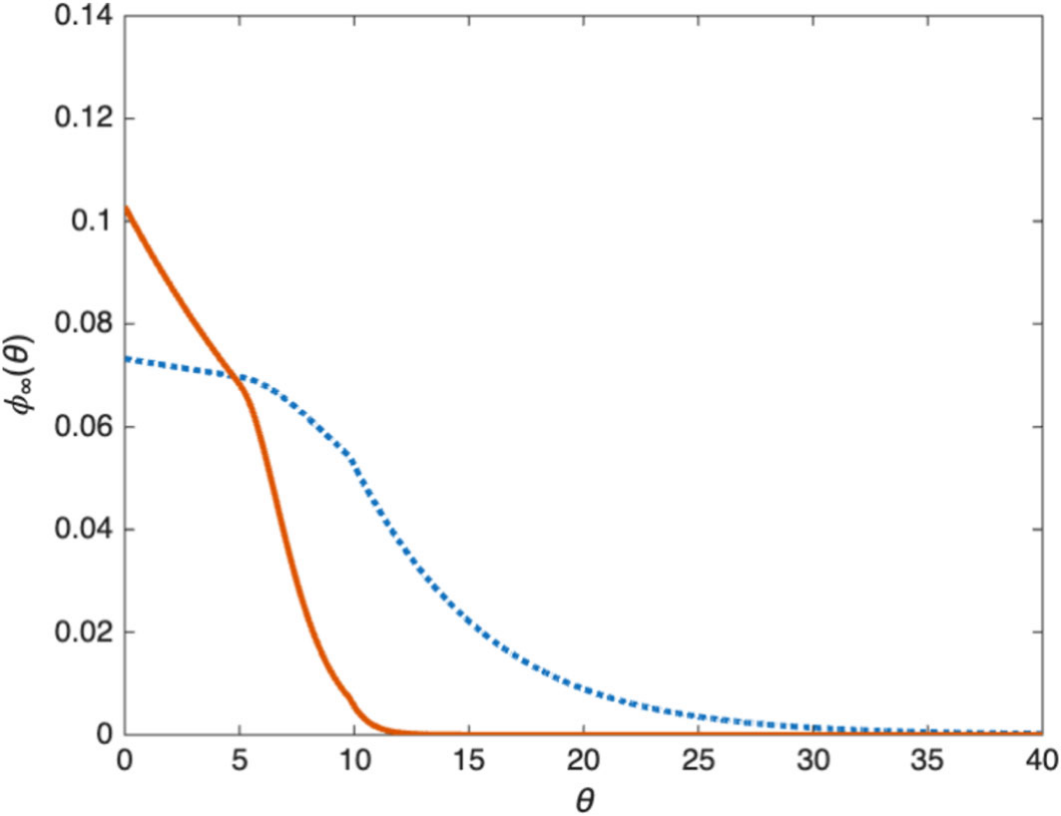
Asymptotic age distribution $\phi _\infty (\theta )$ for Model (I) (dashed blue line) and Model (II) (solid red line) when $\mu$ is given by (3.17)

When $\mu$ is strictly positive for all $\theta$ and (3.15) is satisfied, we can compare the asymptotic age distributions of Model (I) and Model (II): We show in Figure 11 the functions $\phi _\infty (\theta )$ given by Propositions 3.1 (for Model (I)) and  3.2 (for Model (II)) when

3.17 $$\begin{aligned} \mu (\theta )={\left\{ \begin{array}{ll} .01 & \text{ if } \theta <10 \\ .1 & \text{ if } \theta \ge 10 \end{array}\right. } \end{aligned}$$

The slower aging process with Model (II) leads to a very different distribution with very few cells reaching the age $\theta =10$ at which cells begin to die, but also far fewer cells of proliferating age ($\theta \ge 5$) than with Model (I).

### The role of the parameter *m*

Finally, we look at the dependence on the parameter *m*, which appears in the definition of the pressure $p=\frac{m}{m-1} \rho ^{m-1}$. Whenever $m>1$, the degenerate diffusion in the $\rho$ equation (1.6) leads to finite speed of propagation of the support and the behavior as *m* increases is well documented, at least for models without the age variable $\theta$ (see for instance (Perthame et al. 2014a)): the volume density $\rho (x,t)$ develops a sharp front (in the limit $m\rightarrow \infty$, it converges to a characteristic function) while the pressure *p*(*t*, *x*) remains a Lipschitz function with respect to the *x* variable (uniformly in time).

**Fig. 12:**
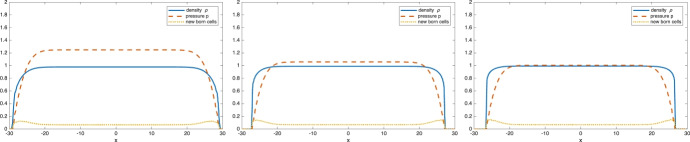
The density $\rho (x,T)$, pressure *p*(*x*, *T*) and distribution of newborn cells at time $T=80$ when $p= \frac{m}{m-1} \rho ^{m-1}$ with m=4 (left), m=8 (middle) and m=12 (right). As *m* increases, the volume density $\rho$ develops a sharp front while the pressure remains Lipschitz

A similar behavior is observed numerically for our model as shown in Figure 12 which gives the volume density, pressure and distribution of new born cells at time $T=80$ for the solution of (1.2) when $\mu (\theta )$ is given by (3.8), $V(\theta )=V_0=1$ and for increasing values of *m*. We note that while the volume density $\rho$ approaches a discontinuous function as *m* increases, the pressure *p* converges to a Lipschitz function. We do not observe any noticeable changes in the distribution of mitosis events (or new born cells), which is consistent with the fact that proliferation is controlled by the pressure (rather than by the volume density).

## Proof of Theorem 2.1

We now turn to the proof of Theorem 2.1. We will prove the existence of a solution for (1.2) in the case where $\mu =0$ in two steps: first we will show that the following regularized system

4.1 $$\begin{aligned} {\left\{ \begin{array}{ll} \partial _t n + \partial _{\theta } n - {\textrm{div}\,}_{x} (n \nabla _{x} p(\rho )) -\varepsilon \Delta _x n = - \nu (\theta ,p) \, n \qquad & x\in \mathbb {R}^d,\; \theta>0, \; t>0\\ \displaystyle n(x,0,t) = 2 \int _0^\infty \nu (\theta ,p)\, n(x,\theta ,t) \, d\theta & x\in \mathbb {R}^d,\; \; t>0\\ n(x,\theta ,0)= n_{in}(x,\theta ) & x\in \mathbb {R}^d,\; \theta >0, \end{array}\right. } \end{aligned}$$

has a solution for all $\varepsilon >0$ where we recall that

$$\begin{aligned} p(\rho ) = \frac{m}{m-1} \rho ^{m-1}, \qquad \rho (x,t) = \int _0^\infty V(\theta ) n(x,\theta ,t)\, d\theta . \end{aligned}$$

Then we will pass to the limit $\varepsilon \rightarrow 0$ to construct a solution of (1.2) and prove our main result.

### Existence of a solution for the regularized system (4.1)

The existence of a solution for (4.1) will be proved by using a discrete time scheme which splits the transport in $\theta$ and the diffusion in *x*. We initialize the scheme by setting $n_0(x,\theta ) = n_{in} (x,\theta )$ and for all $k\ge 0$, we proceed as follows:

**Step 1:** Given $n_k(x,\theta )$, define $n_{k+\frac{1}{2}}(x,\theta )$ solution of

4.2 $$\begin{aligned} {\left\{ \begin{array}{ll} n_{k+\frac{1}{2}} - n_k + \tau \partial _{\theta } n_{k+\frac{1}{2}} = - \tau \nu (\theta ,p_k) \, n_{k+\frac{1}{2}} \qquad & x\in \mathbb {R}^d,\; \theta >0 \\ \displaystyle n_{k+\frac{1}{2}}(x,0) = 2 \int _0^\infty \nu (\theta ,p_k)\, n_k(x,\theta ) \, d\theta & x\in \mathbb {R}^d, \end{array}\right. } \end{aligned}$$

where $p_k = p(\rho _k) = \frac{m}{m-1}\rho _k^{m-1}$ and $\rho _k (x) = \int _0^\infty V(\theta ) n_k(x,\theta )\, d\theta$. Note that the space variable *x* is a parameter in this equation.

**Step 2:** Then define $n_{k+1}(x,\theta ,t)$ solution of

4.3 $$\begin{aligned} n_{k+1} - n_{k+\frac{1}{2}} - \tau {\textrm{div}\,}_{x} (n_{k+1} \nabla _{x} p(\rho _{k+1})) -\varepsilon \tau \Delta n_{k+1}=0\qquad x\in \mathbb {R}^d,\; \theta >0. \end{aligned}$$

Note that the age variable $\theta$ is a parameter in this equation.

This iterative scheme defines $n_k(x,\theta )$ for all $k\in \mathbb {N}$. We then define $n_{\tau }(x,\theta ,t)$ piecewise constant function in *t* by

4.4 $$\begin{aligned} n_{\tau }(x,\theta ,t ) = n_{k}(x,\theta ), \qquad t\in [k\tau ,(k+1)\tau ) \end{aligned}$$

and

$$\begin{aligned} \rho _\tau (x,t)= \int _0^\infty V(\theta ) n_{\tau } (x,\theta ,t) \, d\theta . \end{aligned}$$

We also define the piecewise linear function ${\widetilde{n}}_{\tau }(x,\theta ,t )$ which satisfies ${\widetilde{n}}_{\tau }(x,\theta , k\tau ) = n_k(x,\theta )$:

4.5 $$\begin{aligned} {\widetilde{n}}_{\tau }(x,\theta ,t ) = \left( \frac{(k+1)\tau - t}{\tau }\right) n_k(x,\theta )+ \left( \frac{t-k\tau }{\tau }\right) n_{k+1}(x,\theta ), \qquad t\in [k\tau ,(k+1)\tau ) \end{aligned}$$

and the corresponding volume density

$$\begin{aligned} \widetilde{\rho }_\tau (x,t) =\int _0^\infty {\widetilde{n}}_{\tau } (x,\theta ,t) \, d\theta . \end{aligned}$$

The well-posedness of the scheme (i.e. the existence and uniqueness of a solution to (4.2) and (4.3)) will be proved below. We then note that by combining (4.2) and (4.3), we find

4.6 $$\begin{aligned} {\left\{ \begin{array}{ll} \frac{n_{k+1} - n_k }{\tau } + \partial _{\theta } n_{k+\frac{1}{2}} - {\textrm{div}\,}_{x} (n_{k+1} \nabla _{x} p(\rho _{k+1})) -\varepsilon \Delta n_{k+1}= - \nu (\theta ,p(\rho _k)) \, n_{k+\frac{1}{2}} & x\in \mathbb {R}^d\, \; \theta >0 \\ \displaystyle n_{k+\frac{1}{2}}(x,0) = 2 \int _0^\infty \nu (\theta ,p(\rho _k))\, n_k(x,\theta ) \, d\theta & x\in \mathbb {R}^d. \end{array}\right. } \end{aligned}$$

Our goal is then to derive appropriate bounds on $n_k,\rho _k$ and on the interpolations $n_{\tau }$, $\rho _\tau$ and $\tilde{\rho }_\tau$ in order to pass to the limit $\tau \rightarrow 0$ in (4.6)

### Iterative scheme: existence and estimates from $n_k$, $\rho _k$

The proof of our main result will require several a priori estimates. Throughout these proofs, we use the letter *C* to denote a constant that might change from line to line, but does not depend on *k* and $\tau$.

#### Proposition 4.1

(Well-posedness of Step 1) Given $n_k(x,\theta )$ non-negative function in $L^1(\mathbb {R}^d\times (0,\infty ))$, there exists $n_{k+\frac{1}{2}}(x,\theta )$ solution of (4.2). Furthermore, the volume density satisfies

4.7 $$\begin{aligned} \rho _{k+\frac{1}{2}}(x) \le \frac{1+C \tau }{1-C\tau } \rho _k(x) \qquad \forall x\in \mathbb {R}^d, \end{aligned}$$

4.8 $$\begin{aligned} |\rho _{k+\frac{1}{2}}(x)-\rho _k(x)| \le C\tau \rho _k (x) \qquad \forall x\in \mathbb {R}^d, \end{aligned}$$

for some constant *C* independent of *k* and $\tau$ and for all $\tau >0$ small enough. Finally, we also have

4.9 $$\begin{aligned} \int \varphi (\theta ) [n_{k+\frac{1}{2}}(x,\theta )-n_k(x,\theta )\, d\theta | \le C\Vert \varphi '(\theta )\Vert \rho _k (x) \tau \qquad \forall x\in \mathbb {R}^d. \end{aligned}$$

#### Proof

We rewrite (4.2) as

$$ {\left\{ \begin{array}{ll} (1 + \tau \nu (\theta ,p_k )) n_{k+\frac{1}{2}} + \tau \partial _{\theta } n_{k+\frac{1}{2}} = n_{k} \qquad & \; \theta >0 \\ \displaystyle n_{k+\frac{1}{2}}(x,0) = 2 \int _0^\infty \nu (\theta ,p_k)\, n_k(x,\theta ) \, d\theta . \end{array}\right. } $$

This equation can be solved explicitly: with $A(\theta ) = \int _0^\theta 1 + \tau \nu (\theta ',p_k )\, d\theta ' \ge \theta$, the unique solution of (4.2) is given by:

$$ n_{k+\frac{1}{2}}(x,\theta ) = 2 e^{-\frac{1}{\tau }A(\theta )} \int _0^\infty \nu (\theta ',p_k)\, n_k(x,\theta ') \, d\theta ' + \frac{1}{\tau }\int _0^\theta e^{-\frac{1}{\tau }[A(\theta ) - A(\theta ')] } n_k(x,\theta ') \, d\theta '. $$

Multiplying the equation (4.2) by $V(\theta )$ and integrating with respect to $\theta$, we get the following relation (using (2.3)):

4.10 $$\begin{aligned} \rho _{k+\frac{1}{2}}(x)-\rho _k(x)&= \tau \left[ \int _0^\infty \nu (\theta ,p_k)2V(0) n_k(x,\theta )\, d\theta \right. \nonumber \\&\quad - \int _0^\infty \nu (\theta ,p_k)V(\theta ) n_{k+\frac{1}{2}}(x,\theta )\, d\theta \nonumber \\&\quad +\left. \int _0^\infty V'(\theta ) n_{k+\frac{1}{2}}(x,\theta )\, d\theta \right] \end{aligned}$$

4.11 $$\begin{aligned}&\le C\tau [\rho _k+\rho _{k+\frac{1}{2}}], \end{aligned}$$

which yields first (4.7) and then (4.8). Multiplying the equation (4.2) by $\varphi (\theta )$ and integrating with respect to $\theta$ also gives (4.9). $\square$

#### Proposition 4.2

(Well posedness of Step 2) Given $n_{k+\frac{1}{2}}$ non-negative function in $L^1(\mathbb {R}^d\times (0,\infty ))\cap L^\infty (\mathbb {R}^d;L^1((0,\infty ))$, there exists a non-negative function $n_{k+1}\in L^1(\mathbb {R}^d\times (0,\infty ))\cap L^\infty (\mathbb {R}^d;L^1((0,\infty ))$ solution of (4.3). Furthermore the density satisfies:

4.12 $$\begin{aligned} \int _{\mathbb {R}^d}&\rho _{k+1}^q \, dx +\frac{4 q(q-1)}{(m+q-1)^2} \tau \int _{\mathbb {R}^d}\left| \nabla (\rho _{k+1}^{\frac{m+q-1}{2}})\right| ^2 \, dx +\varepsilon \tau \frac{4(q-1)}{q^2} \int _{\mathbb {R}^d} \left| \nabla \rho _{k+1}^{\frac{q}{2}}\right| ^2\, dx\nonumber \\&\quad \le \int _{\mathbb {R}^d}\rho _{k+\frac{1}{2}}^q\, dx \end{aligned}$$

for all $q\in [1,\infty )$ and

4.13 $$\begin{aligned} \Vert \rho _{k+1} \Vert _{L^\infty (\mathbb {R}^d)} \le \Vert \rho _{k} \Vert _{L^\infty (\mathbb {R}^d)} . \end{aligned}$$

#### Proof

In order to prove that a solution exists, we first notice that by multiplying (4.3) by $V(\theta )$ and integrating with respect to $\theta$, we get

4.14 $$\begin{aligned} \rho _{k+1} - \tau {\textrm{div}\,}_{x} (\rho _{k+1} \nabla _{x} p(\rho _{k+1})) -\tau \varepsilon \Delta \rho _{k+1}=\rho _{k+\frac{1}{2}}\qquad x\in \mathbb {R}^d, \end{aligned}$$

which is a classical nonlinear elliptic equation (recall that $p(\rho ) =\frac{m}{m-1} \rho ^{m-1 }$). Given $\rho _{k+\frac{1}{2}}\in L^1\cap L^\infty (\mathbb {R}^d)$, this equation has a unique solution with $\rho _{k+1}\in L^1\cap L^\infty (\mathbb {R}^d)$. We then define $n_{k+1}(x,\theta )$ the solution of (4.3) with $p (\rho _{k+1}(x))$ given by the solution of (4.14). This solution exists: indeed, $\theta$ is a parameter in this equation, so we only need to solve this equation for a fixed $\theta \ge 0$, and when $\varepsilon >0$, the solution $\rho _{k+1}$ of (4.14) is smooth, so the advection term $\nabla _{x} p(\rho _\varepsilon )$ is smooth and the existence of a solution is straightforward.

Since we first found $\rho _{k+1}(x)$ and then $n_{k+1}(x,\theta )$, we need to make sure that $\int _0^\infty V(\theta ) n_{k+1}(\cdot ,\theta )\, d\theta = \rho _{k+1}$ in order to show that $n_k(x)$ solves (4.3). For that we note that the function $w (x)= \rho _{k+1}(x) - \int _0^\infty V(\theta ) n_{k+1}(x,\theta )\, d\theta$ solves

$$ w - {\textrm{div}\,}(w \nabla p(\rho _{k+1})) - \varepsilon \Delta w=0. $$

In order to prove that $w=0$, we take $\beta _\delta (s)= \sqrt{s^2+\delta ^2}-\delta$ and multiply this equation by $\beta '_\delta (w)$ to find:

$$\begin{aligned} \int w \beta _\delta '(w) \, dx&= - \varepsilon \int \beta _\delta '' |\nabla w|^2\, dx - \int w \beta '_\delta (w) \nabla p(\rho _{k+1}) \cdot \nabla w\, dx\\&\le - \int \nabla p(\rho _{k+1}) \cdot \nabla (w \beta '_\delta (w) - \beta _\delta (w))\, dx\\&\le \int \Delta p(\rho _{k+1}) (w \beta '_\delta (w) - \beta _\delta (w))\, dx. \end{aligned}$$

Since $|s\beta '_\delta (s)-\beta _\delta (s)|\le \delta$ and $s \beta _\delta '(s) \rightarrow |s|$ with $s \beta _\delta '(s) \le |s|$, we deduce $\int |w| \, dx\le 0$, that is, $w= 0$ and so

$$ \int _0^\infty V(\theta ) n_{k+1}(x,\theta )\, d\theta = \rho _{k+1} (x), \qquad \text{ a.e. } x\in \mathbb {R}^d $$

thus proving that $n_k(x)$ is the solution of (4.3).

Next, we derive (4.12). We multiply (4.14) by $\rho _{k+1}^{q-1}$ (for $q>1$) and integrate in *x* to get:

$$\begin{aligned} \int _{\mathbb {R}^d}\rho _{k+1}^{q}\, dx -\tau \rho _{k+1}^{q-1}{\textrm{div}\,}(\rho _{k+1}\nabla p_{k+1})\, dx-\tau \varepsilon \int _{\mathbb {R}^d} \rho _{k+1}^{q-1}\Delta \rho _{k+1} \, dx =\int _{\mathbb {R}^d}\rho _{k+\frac{1}{2}}\rho _{k+1}^{q-1}\,dx. \end{aligned}$$

Rearranging, using the fact that $p_{k+1}=\frac{m}{m-1}\rho _{k+1}^{m-1},$ and using the convexity of the function $s\mapsto s^q$ for any $q >1$ and $s\ge 0$, we deduce:

$$\begin{aligned}&\tau q(q-1)\int _{\mathbb {R}^d}\rho _{k+1}^{m+q-3}|\nabla \rho _{k+1}|^2 \, dx +\tau \varepsilon (q-1)\int _{\mathbb {R}^d}\rho _{k+1}^{q-2}|\nabla \rho _{k+1}|^2 \, dx\\&=\int _{\mathbb {R}^d}\rho _{k+1}^{q-1}(\rho _{k+\frac{1}{2}}-\rho _{k+1})\, dx \\&\le \int _{\mathbb {R}^d} \frac{1}{q} \rho _{k+\frac{1}{2}}^q-\frac{1}{q} \rho _{k+1}^q\, dx. \end{aligned}$$

which implies (4.12). To get (4.13), we can apply the maximum principle to (4.14) or pass to the limit $q\rightarrow \infty$ in the inequality $\Vert \rho _{k+1} \Vert _{L^q(\mathbb {R}^d)} \le \Vert \rho _{k} \Vert _{L^q(\mathbb {R}^d)}$. $\square$

### Estimates for $n_{\tau }$ and $\rho _\tau$

We recall that once the $n_k$ and $\rho _k$ have been iteratively constructed, we can define the piecewise constant functions $n_{\tau }(x,\theta ,t)$ and $\rho _\tau (x,t)$ and the piecewise linear interpolations ${\widetilde{n}}_{\tau }(x,\theta ,t)$, $\widetilde{\rho }_\tau (x,t)$ (see (4.4) and (4.5)). In what follows, we fix $T=K\tau >0$. We start with the following straightforward consequence of Propositions 4.1 and 4.2:

#### Proposition 4.3

There exists *C* such that if $\tau \le \frac{1}{C}$, then $\rho _\tau \ge 0$ is bounded in $L^\infty (0,T;L^q(\mathbb {R}^d))$ for all $q\in [1,\infty ]$ uniformly with respect to $\tau$.

Furthermore, for all $q\in (1,\infty )$, there exists $C_q$ such that if $\tau \le \frac{1}{C_q}$, then

4.15 $$\begin{aligned} \Vert \nabla \rho _{\tau }^{\frac{m+q-1}{2}}\Vert ^2 _{L^2(0,T;L^2(\mathbb {R}^d))} + \varepsilon \Vert \nabla \rho _{\tau }^{\frac{q}{2}}\Vert ^2 _{L^2(0,T;L^2(\mathbb {R}^d))} \le C_q. \end{aligned}$$

#### Proof

Combining (4.12) with (4.7) yields

4.16 $$\begin{aligned} \begin{aligned} \int _{\mathbb {R}^d}\rho _{k+1}^q \, dx&+\frac{4 q(q-1)}{(m+q-1)^2} \tau \int _{\mathbb {R}^d}|\nabla (\rho _{k+1}^{\frac{m+q-1}{2}})|^2 \, dx \\&+\varepsilon \tau \frac{4(q-1)}{q^2} \int _{\mathbb {R}^d} |\nabla \rho _{k+1}^{\frac{q}{2}}|^2\, dx \le \left( \frac{1+C\tau }{1-C\tau }\right) ^q\int _{\mathbb {R}^d}\rho _k^q\, dx. \end{aligned} \end{aligned}$$

Similarly, combining (4.13) with (4.7) yields:

4.17 $$\begin{aligned} \Vert \rho _{k+1} \Vert _{L^\infty (\mathbb {R}^d)} \le \frac{1+C\tau }{1-C\tau } \Vert \rho _{k} \Vert _{L^\infty (\mathbb {R}^d)} . \end{aligned}$$

Using the fact that $\frac{1+x}{1-x}\le 1+4x$ when $x\in [0,1/2]$, and iterating (4.17), we get

$$ \Vert \rho _{K} \Vert _{L^\infty (\mathbb {R}^d)} \le \left( 1+4 C\tau \right) ^K \Vert \rho _{0} \Vert _{L^\infty (\mathbb {R}^d)} \le e^{4CK\tau } \Vert \rho _{0} \Vert _{L^\infty (\mathbb {R}^d)}, $$

as long as $\tau <\frac{1}{2C}$. Similarly, (4.16) gives

$$ \Vert \rho _{K} \Vert _{L^q(\mathbb {R}^d)} \le \left( 1+4 C\tau \right) ^K \Vert \rho _{0} \Vert _{L^q(\mathbb {R}^d)} \le e^{4CK\tau } \Vert \rho _{0} \Vert _{L^q(\mathbb {R}^d)}. $$

These inequalities imply the first statement.

Next, we can rewrite (4.16) as

$$\begin{aligned}&\int _{\mathbb {R}^d}\rho _{k+1}^q \, dx \!+\! \frac{4 q(q-1)}{(m+q-1)^2} \tau \int _{\mathbb {R}^d}|\nabla (\rho _{k+1}^{\frac{m+q-1}{2}})|^2 \, dx \!+\! \varepsilon \tau \frac{4(q-1)}{q^2} \int _{\mathbb {R}^d} |\nabla \rho _{k+1}^{\frac{q}{2}}|^2\, dx \\&\quad \le \int _{\mathbb {R}^d}\rho _k^q\, dx + \left[ \left( \frac{1+C\tau }{1-C\tau }\right) ^q-1\right] \int _{\mathbb {R}^d}\rho _k^q\, dx \\&\quad \le \int _{\mathbb {R}^d}\rho _k^q\, dx + C_q \tau \int _{\mathbb {R}^d}\rho _k^q\, dx, \end{aligned}$$

as long as $C_q\tau \le 1$ for some constant $C_q$ depending on *q*. Summing up for $k=0,\dots K$, we deduce:

$$\begin{aligned} \begin{aligned}&\frac{4 q(q-1)}{(m+q-1)^2} \sum _{k=0}^K \tau \int _{\mathbb {R}^d}|\nabla (\rho _{k+1}^{\frac{m+q-1}{2}})|^2 \, dx \\&\qquad \qquad + \varepsilon \frac{4(q-1)}{q^2} \sum _{k=0}^K \tau \int _{\mathbb {R}^d} |\nabla \rho _{k+1}^{\frac{q}{2}}|^2\, dx \le \left( 1 + C_q K\tau e^{4q CK\tau }\right) \Vert \rho _{0} \Vert ^q_{L^q(\mathbb {R}^d)}, \end{aligned} \end{aligned}$$

which implies (4.15). $$\square $$

Next, we prove the following result, which will be used to control the values of $n_{\tau }$ at infinity (in *x* and $\theta$):

#### Proposition 4.4

There exists a constant *C* such that if $\tau \le \frac{1}{C}$, then

4.18 $$\begin{aligned} \sup _{t\in [0,T]}\int _{\mathbb {R}^d} \int _0^\infty (|x|^2 +\theta ) n_{\tau }(x,\theta ,t) \, d\theta \, dx \le C, \end{aligned}$$

with *C* dependent on *T* by not on $\tau$.

#### Proof

Multiplying (4.2) by $(|x|^2+\theta )\ge 0$ and integrating in *x* and $\theta$ gives

4.19 $$\begin{aligned} &  \int _0^{\infty }\int _{\mathbb {R}^d} (|x|^2+\theta ) n_{k+\frac{1}{2}} \, dx\, d\theta \nonumber \\ &  \quad \le - \tau \int _0^{\infty }\int _{\mathbb {R}^d} \partial _{\theta } n_{k+ \frac{1}{2}}(|x|^2+\theta ) \, dx\,d\theta + \int _0^{\infty }\int _{\mathbb {R}^d} (|x|^2+\theta ) n_{k} \, dx \, d\theta \nonumber \\ &  \quad \le \tau \int _0^{\infty }\int _{\mathbb {R}^d} n_{k+ \frac{1}{2}} \, dx\,d\theta +\tau \int _{\mathbb {R}^d} n_{k+ \frac{1}{2}}(x,0) |x|^2 \, dx \nonumber \\ &  \qquad + \int _0^{\infty }\int _{\mathbb {R}^d} (|x|^2+\theta ) n_{k} \, dx \, d\theta \nonumber \\ &  \quad \le \tau \int _{\mathbb {R}^d} \rho _{k+ \frac{1}{2}} \, dx +\tau \int _{\mathbb {R}^d} 2 \int _0^{\infty } \nu (\theta ,p_k) n_{k} (x,\theta ) |x|^2 \,d\theta \, dx \nonumber \\ &  \qquad + \int _0^{\infty }\int _{\mathbb {R}^d} (|x|^2+\theta ) n_{k} \, dx \, d\theta \nonumber \\ &  \quad \le \tau \int _{\mathbb {R}^d} \rho _{k+ \frac{1}{2}} \, dx + (1+C\tau ) \int _0^{\infty }\int _{\mathbb {R}^d} (|x|^2+\theta ) n_{k} \, dx \, d\theta . \end{aligned}$$

Similarly, (4.3) yields:

$$\begin{aligned} \int _0^{\infty }\int _{\mathbb {R}^d} (|x|^2+\theta ) n_{k+1} \, dx\, d\theta&= \int _0^{\infty }\int _{\mathbb {R}^d} (|x|^2+\theta ) n_{k+\frac{1}{2}} \, dx\, d\theta \\&\quad + 2d \tau \int _{\mathbb {R}^d} \rho _{k+1}^m+ \varepsilon \rho _{k+1}\, dx. \end{aligned}$$

Combining these inequalities give:

$$\begin{aligned} \int _0^{\infty }\int _{\mathbb {R}^d} (|x|^2+\theta ) n_{k+1} \, dx\, d\theta&\le (1+C\tau ) \int _0^{\infty }\int _{\mathbb {R}^d} (|x|^2+\theta ) n_{k} \, dx \, d\theta \\&\quad + C \tau \int _{\mathbb {R}^d} \rho _{k+ \frac{1}{2}} +\rho _{k+1}^m+ \varepsilon \rho _{k+1}\, dx, \end{aligned}$$

and the bounds from Proposition 4.3 (together with (4.7) to control $\rho _{k+ \frac{1}{2}}$) implies

$$ \int _0^{\infty }\int _{\mathbb {R}^d} (|x|^2+\theta ) n_{k+1} \, dx\, d\theta \le (1+C\tau ) \int _0^{\infty }\int _{\mathbb {R}^d} (|x|^2+\theta ) n_{k} \, dx \, d\theta + C(T) \tau , $$

for all $k\le K$ (with $K\tau =T$), which implies

4.20 $$\begin{aligned} \int _0^{\infty }\int _{\mathbb {R}^d} (|x|^2+\theta ) n_{k} \, dx\, d\theta&\le (1+C\tau )^k \int _0^{\infty }\int _{\mathbb {R}^d} (|x|^2+\theta ) n_{in} \, dx\nonumber \\&\quad + C(T) (1+C \tau )^{k+1} \le C (1+C\tau )^k, \end{aligned}$$

and the result follows. $\square$

Finally, we note that while $\rho _\tau$ is bounded in $L^\infty (0,T; L^p(\mathbb {R}^d))$ for $p\in [1,\infty ]$, we only have $n_{\tau }$ bounded in $L^\infty (0,T; L^1(\mathbb {R}^d \times (0,\infty ))$. In order to show that we do not get a measure (in $\theta$) in the limit $\tau \rightarrow 0$, we will make use of the following result:

#### Proposition 4.5

The piecewise constant function $n_{\tau } \ge 0$ is bounded in $L^\infty (0,T;L^1(\mathbb {R}^d\times (0,\infty )))$ and satisfies:

4.21 $$\begin{aligned} \sup _{t\in (0,T)} \int _{\mathbb {R}^d} \int _0^\infty n_{\tau }(x,\theta ,t) \log _+ n_{\tau }(x,\theta ,t) \, d\theta \, dx \le C, \end{aligned}$$

for some constant depending only on *T* and the initial condition.

This proposition follows from the following Lemma which we prove below:

#### Lemma 4.6

There exists a constant *C* such that

4.22 $$\begin{aligned} \begin{aligned}&\int _{\mathbb {R}^d}\int _0^\infty n_{k+1}(x,\theta )\log (n_{k+1}(x,\theta )) V(\theta ) \, d\theta \, dx \\&\qquad + \frac{4\tau }{m}\int _{\mathbb {R}^d} |\nabla (\rho _{k+1})^{m/2}(x)|^2\, dx \\&\quad \le (1+C\tau ) \int _{\mathbb {R}^d}\int _0^\infty n_{k } (x,\theta ) \log (n_{k }(x,\theta )) V(\theta ) \, d\theta \, dx + C \tau (1+C\tau )^k . \end{aligned} \end{aligned}$$

Note that inequality (4.22) also yields a bound on $\nabla \rho _\tau ^{m/2}$. We did not include this estimate in Proposition 4.5 since we will not be using it.

#### Proof of Proposition 4.5

Iterating (4.22), we get:

$$\begin{aligned} \begin{aligned} \int _{\mathbb {R}^d}&\int _0^\infty n_{k}(x,\theta )\log (n_{k}(x,\theta )) V(\theta ) \, d\theta \, dx\\&\quad \le (1+C\tau ) ^k \int _{\mathbb {R}^d}\int _0^\infty n_{in}(x,\theta )\log (n_{in}(x,\theta )) V(\theta ) \, d\theta \, dx \\&\qquad + C k \tau (1+C\tau )^k . \end{aligned} \end{aligned}$$

Recalling that $k\tau = T$, we deduce

$$\begin{aligned} \begin{aligned} \sup _{k=1,\dots , K}&\int _{\mathbb {R}^d}\int _0^\infty n_{k}(x,\theta )\log (n_{k}(x,\theta )) V(\theta ) \, d\theta \, dx\\&\le e^{CT} \int _{\mathbb {R}^d}\int _0^\infty n_{in}(x,\theta )\log (n_{in}(x,\theta )) V(\theta ) \, d\theta \, dx + CT e^{CT}. \end{aligned} \end{aligned}$$

Next, we recall the classical inequality $|s\log s|\chi _{0\le s\le 1} \le s \omega + Ce^{-\omega /2}$ (for all $\omega$) with $\omega = \theta +|x|^2$. We deduce:

$$\begin{aligned}&\int _{\mathbb {R}^d}\int _0^\infty n _\tau \log _+ (n_{\tau }) V(\theta ) \, d\theta \, dx \\&\quad \le \int _{\mathbb {R}^d}\int _0^\infty n _\tau \log (n_{\tau }) V(\theta ) \, d\theta \, dx\\&\qquad + \int _{\mathbb {R}^d}\int _0^\infty \left( (\theta +|x|^2) n _\tau + Ce^{-(\theta +|x|^2)/2} \right) V(\theta ) \, d\theta \, dx, \end{aligned}$$

and so (4.18) implies

4.23 $$\begin{aligned} \int _{\mathbb {R}^d}\int _0^\infty n_{\tau } \log _+ (n_{\tau }) V(\theta ) \, d\theta \, dx \le \int _{\mathbb {R}^d}\int _0^\infty n_{\tau } \log (n_{\tau }) V(\theta ) \, d\theta \, dx + C(T) \end{aligned}$$

for all $t\in [0,T]$ and the result follows. $\square$

#### Proof of Lemma 4.6

Multiplying (4.3) by $V(\theta )( \log (n_{k+1}) +1 )$ and integrating with respect to $\theta$ and *x* yields:

$$ \int _{\mathbb {R}^d} \int _0^\infty (n_{k+1} - n_{k+\frac{1}{2}}) (\log (n_{k+1}) +1) V(\theta ) \, d\theta \, dx +\tau \int _{\mathbb {R}^d} \nabla p(\rho _{k+1}) \cdot \nabla \rho _{k+1} \, dx = 0, $$

and so the convexity of $s\mapsto s\log s$ and the definition of $p(\rho )$ gives

4.24 $$\begin{aligned} \int _{\mathbb {R}^d}\int _0^\infty&n_{k+1} \log (n_{k+1}) V(\theta ) \, d\theta \, dx + \frac{4\tau }{m}\int _{\mathbb {R}^d} |\nabla (\rho _{k+1})^{m/2}|^2\, dx\nonumber \\&\quad \le \int _{\mathbb {R}^d}\int _0^\infty n_{k+\frac{1}{2}} \log (n_{k+\frac{1}{2}}) \, V(\theta ) d\theta \, dx. \end{aligned}$$

Similarly, multiplying (4.2) by $V(\theta )( \log (n_{k+\frac{1}{2}}) +1 )$, we get

$$\begin{aligned}&\int _{\mathbb {R}^d}\int _0^\infty ( n_{k+\frac{1}{2}} - n_k) ( \log (n_{k+\frac{1}{2}}) +1 )V(\theta ) \, d\theta \, dx \\&\quad = -\tau \int _{\mathbb {R}^d}\int _0^\infty \partial _{\theta } ( n_{k+\frac{1}{2}} \log (n_{k+\frac{1}{2}})) V(\theta ) \, d\theta \, dx\\&\qquad - \tau \int _{\mathbb {R}^d}\int _0^\infty \nu (\theta ,p_k) \, n_{k+\frac{1}{2}} ( \log (n_{k+\frac{1}{2}}) +1 )V(\theta ) \, d\theta \, dx \\&\quad \le \tau \int _{\mathbb {R}^d} n_{k+\frac{1}{2}} (x,0)\log (n_{k+\frac{1}{2}} (x,0)) V(0) \, dx\\&\qquad + \tau \int _{\mathbb {R}^d}\int _0^\infty ( n_{k+\frac{1}{2}} \log (n_{k+\frac{1}{2}})) V'(\theta ) \, d\theta \, dx \\&\qquad \qquad - \tau \int _{\mathbb {R}^d}\int _0^\infty \nu (\theta ,p_k) \, n_{k+\frac{1}{2}} \log (n_{k+\frac{1}{2}})V(\theta ) \, d\theta \, dx. \end{aligned}$$

Using the convexity of $s\mapsto s\log s$ again, we deduce

$$\begin{aligned}&\int _{\mathbb {R}^d}\int _0^\infty n_{k+\frac{1}{2}} \log (n_{k+\frac{1}{2}}) V(\theta ) \, d\theta \, dx - \int _{\mathbb {R}^d}\int _0^\infty n_{k } \log (n_{k }) V(\theta ) \, d\theta \, dx \nonumber \\&\qquad \qquad \qquad \le \tau \int _{\mathbb {R}^d} n_{k+\frac{1}{2}} (x,0)\log _+(n_{k+\frac{1}{2}} (x,0)) V(0) \, dx \\&\qquad \qquad \qquad \qquad + \tau \int _{\mathbb {R}^d}\int _0^\infty ( n_{k+\frac{1}{2}} \log (n_{k+\frac{1}{2}})) [ V'(\theta ) -\nu (\theta ,p_k) V(\theta )]\, d\theta \, dx . \end{aligned}$$

The first term can be bounded using the monotonicity of $s\mapsto s\log _+ s$ and the fact that $n_{k+\frac{1}{2}}(x,0)\le C\rho _k(x)$ which is bounded in $L^1\cap L^\infty (\mathbb {R}^d)$ (by a constant depending only on *T*). For the second term, we use the fact that $|V'(\theta ) -\nu (\theta ,p_k) V(\theta )|\le C V(\theta )$ and get:

$$\begin{aligned}&\int _{\mathbb {R}^d}\int _0^\infty n_{k+\frac{1}{2}} \log (n_{k+\frac{1}{2}}) V(\theta ) \, d\theta \, dx - \int _{\mathbb {R}^d}\int _0^\infty n_{k } \log (n_{k }) V(\theta ) \, d\theta \, dx \nonumber \\&\qquad \qquad \le C \tau + C\tau \int _{\mathbb {R}^d}\int _0^\infty \left| n_{k+\frac{1}{2}} \log (n_{k+\frac{1}{2}})) \right| V(\theta )\, d\theta \, dx, \end{aligned}$$

and we can proceed as in (4.23) (we combine (4.19) and (4.20) to get the required bound on the moments of $n_{k+\frac{1}{2}}$) to get:

4.25 $$\begin{aligned}&\int _{\mathbb {R}^d}\int _0^\infty n_{k+\frac{1}{2}} \log (n_{k+\frac{1}{2}}) V(\theta ) \, d\theta \, dx - \int _{\mathbb {R}^d}\int _0^\infty n_{k } \log (n_{k }) V(\theta ) \, d\theta \, dx \nonumber \\&\qquad \le C \tau + C \tau \int _{\mathbb {R}^d}\int _0^\infty ( n_{k+\frac{1}{2}} \log (n_{k+\frac{1}{2}})) V(\theta ) \, d\theta \, dx + C \tau (1+C\tau )^k, \end{aligned}$$

which implies (assuming $\tau$ is small enough so that $C\tau <1/2$ and $\frac{1}{1-C\tau } \le (1+C\tau )$):

4.26 $$\begin{aligned} \int _{\mathbb {R}^d}\int _0^\infty n_{k+\frac{1}{2}} \log (n_{k+\frac{1}{2}}) V(\theta ) \, d\theta \, dx&\le (1+C\tau ) \int _{\mathbb {R}^d}\int _0^\infty n_{k } \log (n_{k }) V(\theta ) \, d\theta \, dx \nonumber \\&\quad + C \tau (1+C\tau )^k. \end{aligned}$$

We combine this inequality with (4.24) to get (4.22). $\square$

### Weak convergence of $n_{\tau }$ and strong convergence of $\rho _\tau$ as $\tau \rightarrow 0$

Since $n_{\tau }$ is bounded in $L^\infty (0,T;L^1(\mathbb {R}^d\times (0,\infty ))$, Propositions 4.4 implies that it converges up to a subsequence for the narrow topology to $n \in L^\infty (0,T;\mathcal {M}(\mathbb {R}^d\times (0,\infty ))$. From now on, we thus fix a subsequence (still denoted $n_{\tau }$ for simplicity) such that

4.27 $$\begin{aligned} \int _0^T \int _{\mathbb {R}^d} \int _0^\infty&n_{\tau }(x,\theta ,t) \psi (x,\theta ,t) \, d\theta \, dx \, dt\nonumber \\&\rightarrow \int _0^T \int _{\mathbb {R}^d} \int _0^\infty n(x,\theta ,t) \psi (t,x,\theta ) \, d\theta \, dx \, dt\end{aligned}$$

for all $\psi \in C^0_b([0,T]\times \mathbb {R}^d\times (0,\infty ))$. Furthermore Proposition 4.5 and Dunford Pettis theorem imply that $n \in L^\infty (0,T;L^1(\mathbb {R}^d\times (0,\infty ))$ and that the limit (4.27) holds for function $\psi \in L^\infty ([0,T]\times \mathbb {R}^d\times (0,\infty ))$. If we take $\psi (x,\theta ,t) = V(\theta ) \phi (x,t)$ in (4.27), we deduce (along the same subsequence)

$$ \rho _\tau (x,t) \rightharpoonup \rho (x,t)= \int _0^\infty n(x,\theta ,t) V(\theta )\, d\theta . $$

In order to pass to the limit $\tau \rightarrow 0$ and derive (4.1), we need to establish the strong convergence of $\rho _\tau$ and $\nabla \rho _\tau ^{m-1}$. A key ingredient for that is the following equation for $\rho _k$, which we obtain by combining (4.10) and (4.14):

4.28 $$\begin{aligned} \rho _{k+1}- \rho _k - \tau {\textrm{div}\,}_{x} (\rho _{k+1} \nabla _{x} p(\rho _{k+1})) -\tau \varepsilon \Delta \rho _{k+1} =\tau F_k \end{aligned}$$

where

$$\begin{aligned} F_k (x)&= \int _0^\infty \nu (\theta ,p_k)2V(0) n_k(x,\theta )\, d\theta - \int _0^\infty \nu (\theta ,p_k)V(\theta ) n_{k+\frac{1}{2}}(x,\theta )\, d\theta \\&\quad + \int _0^\infty V'(\theta ) n_{k+\frac{1}{2}}(x,\theta )\, d\theta . \end{aligned}$$

We have

$$ |F_k(x)| \le C\rho _{k}(x) + C\rho _{k+\frac{1}{2}} (x),$$

and so $F_\tau$ is bounded in $L^\infty (0,T; L^1\cap L^\infty (\mathbb {R}^d))$. We will deduce the following proposition:

#### Proposition 4.7

Up to another subsequence $\rho _\tau (x,t)$ converges strongly to $\rho (x,t)$ in $L^2(0,T;L^2(\mathbb {R}^d))$ and almost everywhere in $[0,T]\times \mathbb {R}^d$.

#### Proof

First, we will prove the convergence of the piecewise linear function $\tilde{\rho }_\tau (t)$: The bound (4.15) with $q=2$ gives

$$\begin{aligned} \varepsilon \Vert \nabla \rho _\tau \Vert _{L^2(0,T;L^2(\mathbb {R}^d))}\le C. \end{aligned}$$

(This bound is the main reason we added the regularization $\varepsilon \Delta n$). Since $\tilde{\rho }_\tau$ is the linear interpolation with the same value as $\rho _\tau$, it is easy to check that we also have

$$\begin{aligned} \varepsilon \Vert \nabla \tilde{\rho }_\tau \Vert _{L^2(0,T;L^2(\mathbb {R}^d)}\le C. \end{aligned}$$

Furthermore, we have

$$ \partial _t \tilde{\rho }_\tau = \frac{\rho _{k+1}- \rho _k }{\tau } = {\textrm{div}\,}_{x} (\rho _{k+1} \nabla _{x} p(\rho _{k+1})) + \varepsilon \Delta \rho _{k+1} +\tau F_k \quad \text{ for } t\in (k\tau ,(k+1)\tau ) $$

and so

$$\begin{aligned} \Vert \partial _t \tilde{\rho }_\tau \Vert _{L^2(0,T;H^{-1}(\Omega ))}\le C. \end{aligned}$$

Together with the bound on $\int _\mathbb {R}^d |x|^2 \tilde{\rho }_\tau \, dx$ (see Proposition 4.4), we have all we need to apply Aubin-Lions and show that $\{ \tilde{\rho }_\tau \}_{\tau >0}$ is pre-compact in $L^2(0,T;L^2(\mathbb {R}^d))$: There exists $\rho \in L^2(0,T;H^1(\mathbb {R}^d))$ such that $\tilde{\rho }_\tau$ converges (up to a subsequence) to $\rho$ strongly in $L^2(0,T;L^2(\mathbb {R}^d))$.

Finally, note that for $t\in [k\tau , (k+1) \tau )$, we have

$$\begin{aligned} \rho _\tau (x,t) - \tilde{\rho }_\tau (x,t)&= \frac{t-k\tau }{\tau } ( \rho _{k+1} -\rho _{k}) \\&= (t-k\tau ) [ {\textrm{div}\,}_{x} (\rho _{k+1} \nabla _{x} p(\rho _{k+1})) +\varepsilon \Delta \rho _{k+1} + F_{k}, \end{aligned}$$

and so

$$ \Vert \rho _\tau -\tilde{\rho }_\tau \Vert _{L^2(0,T;H^{-1}(\mathbb {R}^d))} \le C \tau . $$

It follows that $\rho _\tau$ converges to $\rho$ in $L^2(0,T;H^{-1}(\mathbb {R}^d))$ and since $\rho _\tau$ is bounded in $L^2(0,T;H^{1}(\mathbb {R}^d))$, it also converges strongly in $L^2(0,T;L^2(\mathbb {R}^d))$. $\square$

Finally, since $\rho _\tau$ is bounded in $L^\infty (0,T,L^\infty (\mathbb {R}^d))$, we deduce the convergence of the pressure $p_\tau = \frac{m}{m-1} \rho _\tau ^{m-1}$:

#### Corollary 4.8

Along the same subsequence as above, $\rho _\tau$ converges to $\rho$ strongly in $L^p((0,T)\times \mathbb {R}^d)$ and so $p_\tau (x,t) =\frac{m}{m-1} \rho _\tau (x,t)^{m-1}$ converges to $p(x,t) = \frac{m}{m-1} \rho (x,t)^{m-1}$ strongly in $L^p$ and almost everywhere in $(0,T)\times \mathbb {R}^d$.

### Strong convergence of $\nabla p_\tau$ as $\tau \rightarrow 0$

The strong convergence of $p_\tau$ allows us to pass to the limit in most terms in the weak formulation of (4.1) except for the term $n_{\tau } \nabla p_\tau$. Since $n_{\tau }$ only converges weakly, we have to show that $\nabla p_\tau$ converges strongly. This is proved by using the $\rho _\tau$ equation (4.28) and its limit:

First we define the piecewise constant function

$$F_\tau (x,t) = F_k(x) \qquad t\in [k\tau ,(k+1)\tau ). $$

We note that (4.9) implies

$$\begin{aligned} F_k(x)&= \int _0^\infty \nu (\theta ,p_k)2V(0) n_k(x,\theta )\, d\theta - \int _0^\infty \nu (\theta ,p_k)V(\theta ) n_{k}(x,\theta )\, d\theta \\&\quad + \int _0^\infty V'(\theta ) n_{k}(x,\theta )\, d\theta + \mathcal {O}(C \rho _k(x) \tau ) \end{aligned}$$

with *C* depending on $\Vert V\Vert _\infty$, $\Vert V'\Vert _\infty$, $\Vert \partial _{\theta } \nu \Vert _\infty$ and $\Vert V'' \Vert _\infty$. We can thus write:

$$\begin{aligned} F_\tau (x,t)&= \int _0^\infty \nu (\theta ,p_\tau )2V(0) n_{\tau }(x,\theta ,t)\, d\theta - \int _0^\infty \nu (\theta ,p_\tau )V(\theta ) n_{\tau }(x,\theta ,t)\, d\theta \\&\quad + \int _0^\infty V'(\theta ) n_{\tau }(x,\theta ,t)\, d\theta + \mathcal {O}( \tau ). \end{aligned}$$

The weak convergence of $n_{\tau }$ and the strong convergence of $p_\tau$ imply that $F_\tau$ converges weakly in $L^2(0,T;L^2(\mathbb {R}^d))$ to

$$\begin{aligned} F (x)&= \int _0^\infty \nu (\theta ,p )2V(0) n (x,\theta )\, d\theta - \int _0^\infty \nu (\theta ,p )V(\theta ) n (x,\theta )\, d\theta \\&\quad + \int _0^\infty V'(\theta ) n (x,\theta )\, d\theta . \end{aligned}$$

Furthermore, we can rewrite equation (4.28) as

$$\begin{aligned} \partial _t \tilde{\rho }_\tau - \Delta \rho _\tau ^m - \varepsilon \Delta \rho _\tau = F_\tau \end{aligned}$$

and passing to the limit $\tau \rightarrow 0$ (since $\rho _\tau ^m\rightarrow \rho ^m$) in the sense of distribution, we see that the limit $\rho (x,t)$ solves

4.29 $$\begin{aligned} \partial _t \rho - \Delta \rho ^m -\varepsilon \Delta \rho = F \end{aligned}$$

in $\mathcal {D}'((0,T)\times \mathbb {R}^d)$. This will be the key to proving the following proposition:

#### Proposition 4.9

Up to another subsequence, $\nabla \rho _\tau ^{m-1}$ converges strongly to $\nabla \rho ^{m-1}$.

#### Proof

Multiplying (4.28) by $\rho _k^{m-2}$, and using the convexity of $s\mapsto s^m-1$, we get

$$\begin{aligned}&\int \frac{\rho _{k+1}^{m-1}}{m-1}- \frac{\rho _{k}^{m-1}}{m-1}\, dx + \tau \int \rho _{k+1} \nabla _{x} p(\rho _{k+1}) \nabla \rho _{k+1}^{m-2}\, dx\\&\qquad \qquad \le \int \left( \rho _{k+1}- \rho _k \right) \rho _{k+1}^{m-2}\, dx+ \tau \int \rho _{k+1} \nabla _{x} p(\rho _{k+1}) \nabla \rho _{k+1}^{m-2}\, dx \\&\qquad \qquad \le \tau \int F_k \rho _{k+1}^{m-2}\, dx, \end{aligned}$$

which yields

$$ \int \frac{\rho _{K+1}^{m-1}}{m-1}- \frac{\rho _{0}^{m-1}}{m-1}\, dx +\frac{m-2}{m} \sum _{k=0}^K \tau \int |\nabla _{x} p(\rho _{k+1}) |^2\, dx \le \sum _{k=0}^K \tau \int F_k \rho _{k+1}^{m-2}\, dx, $$

that is

$$ \int \frac{\rho _{\tau }(T)^{m-1}}{m-1}- \frac{\rho _{0}^{m-1}}{m-1}\, dx +\frac{m-2}{m} \int _0^T \int |\nabla _{x} p(\rho _{\tau }) |^2\, dx \le \int _0^T \int F_\tau \rho _{\tau }^{m-2}\, dx + \mathcal {O}(\tau ), $$

so that

$$\begin{aligned}\liminf _{\tau \rightarrow 0}\frac{m-2}{m} \int _0^T \int |\nabla _{x} p(\rho _{\tau }) |^2\, dx&\le - \int \frac{\rho (T)^{m-1}}{m-1}\, dx + \int \frac{\rho _{0}^{m-1}}{m-1}\, dx\\&\quad + \int _0^T \int F \rho ^{m-2}\, dx . \end{aligned}$$

We now use the limiting equation for $\rho$, (4.29). Multiplying that equation by $\rho ^{m-2}$ and integrating in *x* and *t* gives

$$ \int \frac{\rho (T)^{m-1}}{m-1}- \frac{\rho _{0}^{m-1}}{m-1}\, dx +\frac{m-2}{m} \int _0^T \int |\nabla _{x} p(\rho ) |^2\, dx = \int _0^T \int F \rho ^{m-2}\, dx. $$

Together, these two relations imply

$$\liminf _{\tau \rightarrow 0}\frac{m-2}{m} \int _0^T \int |\nabla _{x} p(\rho _{\tau }) |^2\, dx \le \frac{m-2}{m} \int _0^T \int |\nabla _{x} p(\rho ) |^2\, dx, $$

which in turn means that $\nabla _{x} p(\rho _\tau )$ converges strongly to $\nabla _{x} p(\rho )$ in $L^2(0,T;L^2(\mathbb {R}^d))$. $\square$

### The limit $\tau \rightarrow 0$: solutions of (4.1)

Given a smooth test function $\varphi (x,\theta ,t)$, compactly supported in $\mathbb {R}^d\times [0,\infty )\times [0,\infty )$, we multiply (4.6) by $\varphi _{k}=\varphi (x,\theta ,k\tau )$, integrate with respect to *x* and $\theta$ and sum over *k*:

$$\begin{aligned}&\sum _{k=0}^\infty \tau \int _{\mathbb {R}^d} \int _0^\infty \frac{1}{\tau } ( n_{k+1}\varphi _k - n_k\varphi _k) \\&\qquad - n_{k+\frac{1}{2}} \partial _{\theta } \varphi _k + n_{k+1} \nabla _{x} p(\rho _{k+1}) \cdot \nabla _{x} \varphi _k - \varepsilon n_{k+1} \Delta _x \varphi _k \, d\theta \, dx \\&\quad = - \sum _{k=0}^\infty \tau \int _{\mathbb {R}^d} 2 \int _0^\infty \nu (\theta ,p_k)\, n_k(x,\theta ) \, d\theta \varphi _k(0)\, dx\\&\qquad - \sum _{k=0}^\infty \tau \int _{\mathbb {R}^d} \int _0^\infty \nu (\theta ,p_k) \, n_{k+\frac{1}{2}} \varphi _k\, d\theta \, dx , \end{aligned}$$

and writing $\sum _{k=0}^\infty n_{k+1}\varphi _k - n_k\varphi _k =\sum _{k=1}^\infty n_{k}(\varphi _{k-1} - \varphi _k) - n_0\varphi _0$, we deduce:

$$\begin{aligned}&\int _0^\infty \int _{\mathbb {R}^d} \int _0^\infty - n_{\tau } \partial _t \varphi - \bar{n}_\tau \partial _{\theta } \varphi + n_{\tau } \nabla _{x} p(\rho _\tau ) \cdot \nabla _{x} \varphi \, d\theta - \varepsilon n_{\tau } \Delta _x \varphi \, dx\, dt \\&\quad = - \int _0^\infty \int _{\mathbb {R}^d} 2 \int _0^\infty \nu (\theta ,p_\tau )\, n_{\tau }(x,\theta ,t) \, d\theta \varphi (x,0,t)\, dx\, dt\\&\qquad - \int _0^\infty \int _{\mathbb {R}^d} \int _0^\infty \nu (\theta ,p_\tau ) \,\bar{n}_\tau \varphi \, d\theta \, dx \, dt+ \mathcal {O}(\tau ), \end{aligned}$$

where $\bar{n}_\tau$ is the piecewise constant function equal to $n_{k+\frac{1}{2}}$ on the interval $[k\tau ,(k+1)\tau )$.

We note (see (4.19) and (4.26)) that $n_{k+\frac{1}{2}}$ satisfies the same bounds on $n_k$ so that $\bar{n}_\tau (x,\theta ,t)$ converges weakly in $L^1(0,T;L^1(\mathbb {R}^d\times (0,\infty ))$ as well (up to another subsequence) and (4.9) implies that this limit must be $n(x,\theta ,t)$ (and therefore that the original subsequence converges).

We can thus pass to the limit in all the terms. For the diffusion term, we have to write

$$ \int _0^\infty \int _{\mathbb {R}^d} \int _0^\infty n_{\tau } \nabla _{x} p(\rho _\tau ) \cdot \nabla _{x} \varphi \, d\theta \, dx\, dt = \int _0^\infty \int _{\mathbb {R}^d} \int _0^\infty n_{\tau } \nabla _{x} \varphi \, d\theta \cdot \nabla _{x} p(\rho _\tau ) \, dx\, dt , $$

and note that $\int _0^\infty n_{\tau } \nabla _{x} \varphi \, d\theta$ converges weakly in $L^2(0,T;L^2(\mathbb {R}^d))$ to $\int _0^\infty n \nabla _{x} \varphi \, d\theta$ in order to pass to the limit in that term (using the fact that $\nabla _{x} p(\rho _\tau )$ converges strongly).

We deduce that $n(x,\theta ,t)$ is a weak solution of (4.1) in the sense that

4.30 $$\begin{aligned} &  \int _0^\infty \int _{\mathbb {R}^d} \int _0^\infty - n \partial _t \varphi - n \partial _{\theta } \varphi + n \nabla _{x} p(\rho ) \cdot \nabla _{x} \varphi \, d\theta - \varepsilon n \Delta _x \varphi \, dx\, dt \nonumber \\ &  \quad = - \int _0^\infty \int _{\mathbb {R}^d} 2 \int _0^\infty \nu (\theta ,p)\, n(x,\theta ,t) \, d\theta \varphi (x,0,t)\, dx\, dt \nonumber \\ &  \qquad - \int _0^\infty \int _{\mathbb {R}^d} \int _0^\infty \nu (\theta ,p) \,\bar{n} \varphi \, d\theta \, dx \, dt. \end{aligned}$$

### The limit $\varepsilon \rightarrow 0$: proof of Theorem 2.1

From now on, we denote by $n_\varepsilon (x,\theta ,t)$ the solution of (4.1) constructed in the previous section and by $\rho _\varepsilon (x,t)$ the corresponding volume density. We saw that $n_\varepsilon$, $\rho _\varepsilon$ satisfies (4.30) and we now need to pass to the limit $\varepsilon \rightarrow 0$.

Much of the arguments are similar to the limit $\tau \rightarrow 0$. Indeed, Proposition 4.3 implies that $\rho _\varepsilon$ is bounded in $L^\infty (0,T;L^q(\mathbb {R}^d))$ for all $q\in [1,\infty ]$ and by passing to the limit $\tau \rightarrow 0$ in (4.15), (4.18) and (4.21), we easily get the following bounds (uniform in $\varepsilon$):

4.31 $$\begin{aligned} \Vert \nabla \rho _{\varepsilon }^{\frac{m+q-1}{2}}\Vert ^2 _{L^2(0,T;L^2(\mathbb {R}^d))}\le C_q \qquad \forall q>1, \end{aligned}$$

4.32 $$\begin{aligned} \sup _{t\in [0,T]}\int _{\mathbb {R}^d} \int _0^\infty (|x|^2 +\theta ) n_\varepsilon (x,\theta ,t) \, d\theta \, dx \le C, \end{aligned}$$

4.33 $$\begin{aligned} \int _{\mathbb {R}^d} \int _0^\infty \left| n_\varepsilon \log n_\varepsilon \right| \, dx \, d\theta \le C. \end{aligned}$$

In particular, we can proceed as Section 4.4 to prove the existence of a subsequence $\varepsilon \rightarrow 0$ along which

$$\begin{aligned} \int _0^T \int _{\mathbb {R}^d} \int _0^\infty&n_\varepsilon (x,\theta ,t) \psi (x,\theta ,t) \, d\theta \, dx \, dt\\&\rightarrow \int _0^T \int _{\mathbb {R}^d} \int _0^\infty n(x,\theta ,t) \psi (t,x,\theta ) \, d\theta \, dx \, dt \end{aligned}$$

for all $\psi \in L^\infty ([0,T]\times \mathbb {R}^d\times (0,\infty ))$ and

$$ \rho _\varepsilon (x,t) \rightharpoonup \rho (x,t)= \int _0^\infty n(x,\theta ,t) V(\theta )\, d\theta . $$

The main difference is in the way we prove the strong convergence of $\rho _\varepsilon$, since in the proof of Proposition 4.7, we used the bound on $\nabla \rho _\tau$ which was not uniform in $\varepsilon$. Instead, we will use (4.31) to prove the strong convergence of $\rho _\varepsilon ^s$ for *s* large enough (the argument below does not appear to work with the time approximation, which is the reason we had to introduce the regularization $\varepsilon >0$ in the system):

#### Proposition 4.10

The sequence $\rho _\varepsilon ^{m+2}$ is precompact in $L^2(0,T;L^2(\mathbb {R}^d))$.

We immediately note that this implies the existence of a subsequence along which $\rho _\varepsilon ^{m+2}$ converges strongly in $L^2$ and almost everywhere. This implies the convergence almost everywhere of $\rho _\varepsilon$ and the uniform bounds in $L^\infty (0,T;L^q(\mathbb {R}^d))$ implies that $\rho _\varepsilon$ converges strongly in $L^q(0,T;L^q(\mathbb {R}^d))$ for all $q\in [1,\infty )$.

We can then prove the strong convergence of $\nabla p_\varepsilon = \frac{m}{m-1}\nabla \rho _\varepsilon ^{m-1}$ as in Proposition 4.9 and pass to the limit in (4.30) to get (2.6) and complete the proof of Theorem 2.1.

#### Proof of Proposition 4.10

Taking $q=m+5$ in (4.31) implies that $\rho _\varepsilon ^{m+2}$ is bounded in $L^2(0,T;H^1(\mathbb {R}^d))$. Furthermore, we recall that $\rho _\varepsilon$ solves (see (4.29))

$$ \partial _t \rho _\varepsilon -\Delta \rho _\varepsilon ^m -\varepsilon \Delta \rho _\varepsilon = F_\varepsilon $$

with $F_\varepsilon$ bounded in $L^2(0,T;L^2(\mathbb {R}^d))$ and so

$$\begin{aligned} \frac{1}{m+2} \partial _t \rho _\varepsilon ^{m+2}&= \rho _\varepsilon ^{m+1} \Delta \rho _\varepsilon ^m + \varepsilon \rho _\varepsilon ^{m+1} \Delta \rho _\varepsilon + F_\varepsilon \rho _\varepsilon ^{m-1}. \end{aligned}$$

To show that the right-hand-side is bounded, we rewrite

$$\begin{aligned} \rho _\varepsilon ^{m+1} \Delta \rho _\varepsilon ^m = {\textrm{div}\,}(\rho _\varepsilon ^{m+1} \nabla \rho _\varepsilon ^m) - \nabla \rho _\varepsilon ^{m+1}\cdot \nabla \rho _\varepsilon ^m \end{aligned}$$

and

$$ \rho _\varepsilon ^{m+1} \Delta \rho _\varepsilon ={\textrm{div}\,}( \rho _\varepsilon ^{m+1} \nabla \rho _\varepsilon ) - \nabla \rho _\varepsilon ^{m+1} \cdot \nabla \rho _\varepsilon =\frac{1}{m+2}\Delta \rho _\varepsilon ^{m+2} - \frac{2(m+1)}{(m+2)^2}\left| \nabla \rho _\varepsilon ^{\frac{m+2}{2}} \right| ^2. $$

Since (4.31) implies that $\nabla \rho _\varepsilon ^s$ is bounded in $L^2(0,T;L^2(\mathbb {R}^d))$ for all $s>\frac{m}{2}$, we see that these two terms are bounded in $L^2(0,T;H^{-1}(\mathbb {R}^d))+L^1(0,T;L^1(\mathbb {R}^d))$.

We have thus shown that $\rho _\varepsilon ^{m+2}$ is bounded in $L^2(0,T;H^1(\mathbb {R}^d))$ and that $\partial _t \rho _\varepsilon ^{m+2}$ is bounded in $L^2(0,T;H^{-1}(\mathbb {R}^d))+L^1(0,T;L^1(\mathbb {R}^d))$. Together with (4.32), this is enough to apply Aubin-Lions lemma to prove that $\rho _\varepsilon ^{m+2}$ is precompact in $L^2(0,T;L^2(\mathbb {R}^d))$. $\square$

## Figures and Tables

**Algorithm 1:**
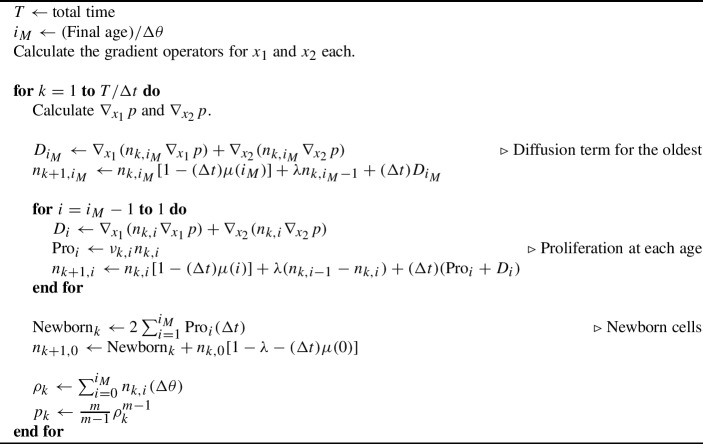
Pseudocode for the simulation

## Data Availability

Data sharing is not applicable to this article (no datasets were generated or analyzed during the study).

## Appendix A Numerical scheme and algorithm

Focusing on the discretization of the variables $\theta$ and *t*, we write $n_{k,i}(x) = n(x,k \Delta t, i \Delta \theta )$. Using first-order approximations, Equation (1.2) leads to

A.1 $$\begin{aligned} \frac{n_{k+1,i}-n_{k,i}}{\Delta t} + \frac{n_{k,i}-n_{k,i-1}}{\Delta \theta } - {\textrm{div}\,}_{x} (n_{k,i} \nabla _{x} p_{k}) = -\nu _{k,i} n_{k,i} -\mu _{i} n_{k,i}, \end{aligned}$$

for $i=1,\dots , i_M-1$. Setting $\lambda :=\Delta t / \Delta \theta$, and rearranging (A.1), we obtain

A.2 $$\begin{aligned} n_{k+1,i} = n_{k,i} + \lambda (n_{k,i-1}-n_{k,i}) + {\textrm{div}\,}_{x} (n_{k,i}\nabla _{x} p_k)\Delta t - \nu _{k,i} n_{k,i}\Delta t -\mu _{i}n_{k,i}\Delta t. \end{aligned}$$

Since cells that reach the age $\theta _{max} = i_M \Delta \theta$ do not age or duplicate, the equation for $n_{k+1,i_M}$ is:

A.3 $$\begin{aligned} n_{k+1,i_{M}} = n_{k,i_{M}} + \lambda n_{k,i_M-1} + (\Delta t) [{\textrm{div}\,}_{x} (n_{k,i_M}\nabla _{x} p_k) - \nu _{k,i_M} n_{k,i_M}-\mu _{i_M}n_{k,i_M}] . \end{aligned}$$

The density at time $k\Delta t$ is then given by

$$\begin{aligned} \rho _k = \sum _{i=0}^{i_M} n_{k,i} \Delta \theta , \end{aligned}$$

and using (A.1)-(A.2), we get

A.4 $$\begin{aligned} \rho _{k+1} - n_{k+1,0} \Delta \theta&= \rho _k-n_{k,0}\Delta \theta + n_{k,0}\Delta t\nonumber \\&\quad + (\Delta t\Delta \theta )\sum _{i=1}^{i_M}[{\textrm{div}\,}_{x}(n_{k,i}\nabla _{x} p_k)-\nu _{k,i}n_{k,i}-\mu _in_{k,i}]. \end{aligned}$$

With a slight abuse of notation, we write $\nu _k\rho _k = \sum _{i=0}^{i_M}\nu _{k,i}n_{k,i}\Delta \theta$ and $\mu \rho _k = \sum _{i=0}^{i_M}\mu _in_{k,i}\Delta \theta ,$ so that equation (A.4) can be rewritten as

A.5 $$\begin{aligned} \rho _{k+1}&= \rho _k +(n_{k+1,0}-n_{k,0})\Delta \theta + n_{k,0}\Delta t + {\textrm{div}\,}_{x} (\rho _k\nabla _{x}p_k)\Delta t \nonumber \\&\quad - {\textrm{div}\,}_{x} (n_{k,0}\nabla _{x}p_k)\Delta t\Delta \theta \nonumber \\&\quad - \nu _k\rho _k\Delta t +\nu _{k,0}n_{k,0}\Delta t\Delta \theta -\mu \rho _k \Delta t +\mu _0n_{k,0}\Delta t\Delta \theta . \end{aligned}$$

With the same notation, the equation for the density (1.7) can be discretized as $\rho _{k+1}=\rho _k+{\textrm{div}\,}_{x}(\rho _k\nabla _{x}p_k)\Delta t+\nu _k\rho _k\Delta t-\mu \rho _k\Delta t$. Combining these two equations leads to

$$\begin{aligned} 2\nu _k\rho _k\Delta t&= (n_{k+1,0}-n_{k,0})\Delta \theta +n_{k,0}\Delta t - {\textrm{div}\,}_{x} (n_{k,0}\nabla _{x}p_k)\Delta t\Delta \theta + \nu _{k,0}n_{k,0}\Delta t\Delta \theta \nonumber \\&\quad +\mu _0n_{k,0}\Delta t\Delta \theta , \end{aligned}$$

which gives the appropriate discretization of the condition at $\theta =0$ ($i=0$):

A.6 $$\begin{aligned} n_{k+1,0}= &  2\sum _{i=0}^{i_M}\nu _{k,i}n_{k,i}\Delta t +n_{k,0}\left( 1-\lambda \right) +{\textrm{div}\,}_{x}(n_{k,0}\nabla _{x} p_k)\Delta t - \nu _{k,0}n_{k,0}\Delta t -\mu _0n_{k,0}\Delta t \nonumber \\= &  2\sum _{i=1}^{i_M}\nu _{k,i}n_{k,i}\Delta t + n_{k,0}\left( 1-\lambda +\nu _{k,0}\Delta t-\mu _0\Delta t\right) + {\textrm{div}\,}_{x}(n_{k,0}\nabla _{x} p_k)\Delta t. \nonumber \\ \end{aligned}$$

Equations (A.2)-(A.3)-(A.6) provide the numerical scheme for the aging/duplication/death of the cells. Importantly, we note that in order to reduce the computational cost, it might be necessary to take $\Delta \theta$ to be much larger than $\Delta t$ ($\lambda \ll 1$). The discretization with respect to the spatial variable *x* is done with a uniform grid $\Delta x_1= \Delta x_2 = \Delta x$ and the gradients are calculated by the centered difference

$$\begin{aligned} (\nabla _{x_i} p)_{l,m} = \frac{p_{l+1,m}-p_{l-1,m}}{2\Delta x}, \qquad i=1,2. \end{aligned}$$

Algorithm 1 provides the pseudocode for the algorithm used for the simulation.
